# Red Blood Cell and Endothelial eNOS Independently Regulate Circulating Nitric Oxide Metabolites and Blood Pressure

**DOI:** 10.1161/CIRCULATIONAHA.120.049606

**Published:** 2021-07-07

**Authors:** Francesca Leo, Tatsiana Suvorava, Sophia K. Heuser, Junjie Li, Anthea LoBue, Frederik Barbarino, Eugenia Piragine, Rebekka Schneckmann, Beate Hutzler, Miranda E. Good, Bernadette O. Fernandez, Lukas Vornholz, Stephen Rogers, Allan Doctor, Maria Grandoch, Johannes Stegbauer, Eddie Weitzberg, Martin Feelisch, Jon O. Lundberg, Brant E. Isakson, Malte Kelm, Miriam M. Cortese-Krott

**Affiliations:** Myocardial Infarction Research Laboratory, Department of Cardiology, Pulmonology, and Angiology (F.L., T.S., S.K.H., J.L., A.L.B., F.B., E.P., B.H., L.V., M.M.C.-K.), Medical Faculty, Heinrich-Heine-University, Düsseldorf, Germany.; Department of Pharmacology and Clinical Pharmacology (R.S., M.G.), Medical Faculty, Heinrich-Heine-University, Düsseldorf, Germany.; Department of Nephrology (J.S.), Medical Faculty, Heinrich-Heine-University, Düsseldorf, Germany.; Department of Cardiology Pneumology and Angiology (T.S., M.K., M.M.C.-K.), Medical Faculty, Heinrich-Heine-University, Düsseldorf, Germany.; CARID, Cardiovascular Research Institute Düsseldorf (M.K.), Medical Faculty, Heinrich-Heine-University, Düsseldorf, Germany.; Department of Pharmacy, University of Pisa, Italy (F.P.).; Robert M. Berne Cardiovascular Research Center, Department of Molecular Physiology and Biophysics, University of Virginia School of Medicine, Charlottesville (M.E.G., B.E.I.).; Molecular Cardiology Research Institute, Tufts Medical Center, Boston, MA (M.E.G.).; Clinical & Experimental Sciences, Faculty of Medicine, University of Southampton, United Kingdom (B.O.F.).; Department of Pediatrics, Center for Blood Oxygen Transport and Hemostasis, University of Maryland School of Medicine, Baltimore (S.R., A.D.).; Department of Physiology and Pharmacology, Karolinska Institute, Stockholm, Sweden (E.W., J.O.L., M.M.C.-K.).

**Keywords:** blood circulation, blood pressure, hypertension, models, animal, nitric oxide synthase

## Abstract

Current paradigms suggest that nitric oxide (NO) produced by endothelial cells (ECs) through endothelial nitric oxide synthase (eNOS) in the vessel wall is the primary regulator of blood flow and blood pressure. However, red blood cells (RBCs) also carry a catalytically active eNOS, but its role is controversial and remains undefined. This study aimed to elucidate the functional significance of RBC eNOS compared with EC eNOS for vascular hemodynamics and nitric oxide metabolism.

**Methods:**

We generated tissue-specific loss- and gain-of-function models for eNOS by using cell-specific Cre-induced gene inactivation or reactivation. We created 2 founder lines carrying a floxed eNOS (eNOS^flox/flox^) for Cre-inducible knockout (KO), and gene construct with an inactivated floxed/inverted exon (eNOS^inv/inv^) for a Cre-inducible knock-in (KI), which respectively allow targeted deletion or reactivation of eNOS in erythroid cells (RBC eNOS KO or RBC eNOS KI mice) or in ECs (EC eNOS KO or EC eNOS KI mice). Vascular function, hemodynamics, and nitric oxide metabolism were compared ex vivo and in vivo.

**Results:**

The EC eNOS KOs exhibited significantly impaired aortic dilatory responses to acetylcholine, loss of flow-mediated dilation, and increased systolic and diastolic blood pressure. RBC eNOS KO mice showed no alterations in acetylcholine-mediated dilation or flow-mediated dilation but were hypertensive. Treatment with the nitric oxide synthase inhibitor *N*^γ^-nitro-l-arginine methyl ester further increased blood pressure in RBC eNOS KOs, demonstrating that eNOS in both ECs and RBCs contributes to blood pressure regulation. Although both EC eNOS KOs and RBC eNOS KOs had lower plasma nitrite and nitrate concentrations, the levels of bound NO in RBCs were lower in RBC eNOS KOs than in EC eNOS KOs. Reactivation of eNOS in ECs or RBCs rescues the hypertensive phenotype of the eNOS^inv/inv^ mice, whereas the levels of bound NO were restored only in RBC eNOS KI mice.

**Conclusions:**

These data reveal that eNOS in ECs and RBCs contribute independently to blood pressure homeostasis.

## Abstract 2

Supplemental Digital Content is available in the text.

**Clinical Perspective**

**What Is New?**

- We generated endothelial cell– and red blood cell (RBC)-specific endothelial nitric oxide synthase (eNOS) knockout and eNOS knock-in mice
- Endothelial cell eNOS knockout mice show hypertension, endothelial dysfunction, and increased systemic vascular resistance, whereas reactivation of eNOS in endothelial cells restores endothelial function and normotension.
- RBC eNOS knockout mice show hypertension, a preserved arterial endothelial function, and reduced levels of bound nitric oxide in RBCs, whereas reactivation of eNOS in RBCs restores nitric oxide bioavailability in RBCs and rescues the hypertensive phenotype.

**What Are the Clinical Implications?**

- Both endothelial cells and RBCs are important regulators of blood pressure through eNOS.
- RBC eNOS contributes to the regulation of nitric oxide metabolism, systemic hemodynamics, and blood pressure.
- These findings may have important pathophysiological implications in our understanding of the interrelationship between hematologic and cardiovascular disease and may reveal novel therapeutic approaches to improve tissue perfusion.

**Editorial, see p 890**

Nitric oxide (NO) produced by the endothelial isoform of nitric oxide synthase (eNOS) in endothelial cells (ECs) is considered to be the central regulator of vascular tone and systemic hemodynamics.^1^ All strains of global eNOS knockout (KO) mice are hypertensive,^2–4^ and some show decreased levels of the circulating nitric oxide (NO) oxidation products, nitrite and nitrate.^5–7^

However, eNOS is also expressed in other cell types, including red blood cells (RBCs).^8,9^ The functional significance of eNOS in RBC physiology, systemic NO metabolism, and tissue protection remains controversial.^10–16^ There are indications that eNOS in the blood may participate in the regulation of circulating nitrite levels and blood pressure (BP) homeostasis,^9^ but its specific contribution is unknown. One way to test the physiological effect directly would be through genetic manipulation of eNOS in ECs versus RBCs.

This study aimed at elucidating the specific role of eNOS in RBCs in direct comparison with the role of eNOS in ECs in controlling systemic NO metabolism and BP regulation. To accomplish this, we generated tissue-specific loss- and gain-of-function models for eNOS by using tissue-specific Cre-induced gene inactivation or reactivation. To our knowledge, these studies are the first performed on tissue-specific eNOS KO/knock-in (KI) mice providing compelling evidence that eNOS in RBCs contributes to the regulation of systemic NO bioavailability and systemic hemodynamics, independently of eNOS in the endothelium. Altogether, these findings suggest the existence of a noncanonical RBC eNOS-dependent pathway for regulation of BP homeostasis independent of the eNOS expressed in the vessel wall.

## Methods

A detailed description of the methods is available in the Data Supplement. The data that support the findings of this study are available from the corresponding author on reasonable request.

### Materials

Unless otherwise specified, chemicals were purchased from Sigma-Aldrich Co LLC.

### Animals

All experiments were approved by the Landesamt für Natur, Umwelt und Verbraucherschutz (LANUV) according to the European Convention for the Protection of Vertebrate Animals used for Experimental and other Scientific Purposes (Council of Europe Treaty Series No. 123). Animal care was provided according to the institutional guidelines. Tamoxifen-inducible endothelial-specific Cre mice (Tg(Cdh5-Cre/ERT2)1Rha; MGI:3848982)^17^ were kindly provided by Prof Dr E. Lammert (Heinrich-Heine-University of Düsseldorf). Mice expressing Cre recombinase in erythroid cells under the control of the promoter of the hemoglobin β-chain (C57BL/6-Tg(Hbb-Cre)12Kpe/J; MGI: J:89725)^18^ were obtained by Jackson Laboratory (JAX stock No. 008314) and crossed for >10 generations with C57BL/6J. DeleterCre (C57Bl/6.C-Tg(CMVCre)1Cgn/J)^19^ mice expressing Cre in all tissues were kindly provided by Prof Claus Pfeffer (Heinrich Heine University of Düsseldorf). Experimental planning and execution followed the ARRIVE recommendations (Animal Research: Reporting of In Vivo Experiments).^20^ For experiments, 2- to 6-months-old male mice up to 30 g were used. Mice of the same genotype and age were randomly assigned to the experimental groups. The evaluation of data obtained by ultrasound was performed by a blinded researcher.

### Generation of EC/RBC eNOS KO and EC/RBC eNOS KI Mice

We generated 2 independent founder lines carrying a floxed eNOS (eNOS^flox/flox^) or a gene construct with an inactivated floxed/inverted exon (eNOS^inv/inv^) for a Cre-inducible KI. Phenotypically eNOS^flox/flox^ are wild-type (WT) mice, and eNOS^inv/inv^ are conditional global eNOS KO (CondKO) mice. These founder lines respectively allow targeted removal or reactivation of eNOS in either ECs or RBCs, or all cells. To generate eNOS^flox/flox^ mice, we designed a loxP eNOS targeting construct by simultaneously inserting an orphan loxP site and an FRT-neo-FRT-loxP resistance cassette inserted into the Nos3 genomic locus to target exon 2 of Nos3 by Cre-mediated excision. To generate eNOS^inv/inv^ mice, we inserted an inverted exon 2 of Nos3 and 2 additional Lox511 sites in the loxP-eNOS construct, which allowed the Cre-induced reactivation of eNOS in a cell type of interest. The plasmids were sequenced, linearized, and electroporated in A9 embryonic stem cells (hybrid C57/129), 300 clones were picked, and positive clones were screened by Southern blot at the 5′ arm and by long-range polymerase chain reaction (PCR). Homozygous eNOS^flox/flox^ mice or eNOS^inv/inv^ mice were crossed with Cdh5-Cre/ERT2^pos^ mice to obtain eNOS^flox/flox^ Cdh5-Cre/ERT2^pos^ and eNOS^flox/flox^ Cdh5-Cre/ERT2^neg^ mice or eNOS^inv/inv^ Cdh5-Cre/ERT2^pos^ and eNOS^inv/inv^ Cdh5-Cre/ERT2^neg^ mice, respectively. To induce EC-specific activation of the Cre recombinase, we treated Cre-positive and Cre-negative mice of each line with tamoxifen (33 mg·kg^–1^·d^–1^) for 5 consecutive days and allowed a 21-day waiting period after the last injection, which generated EC eNOS KO (eNOS^flox/flox^ Cdh5-Cre/ERT2^pos^+TAM) mice and EC eNOS KI (eNOS^inv/inv^ Cdh5-Cre/ERT2^pos^+TAM) mice and their respective Cre-negative controls. Homozygous eNOS^flox/flox^ mice or eNOS^inv/inv^ mice were also crossed with erythroid-specific Hbb-Cre^pos^ mice to obtain erythroid-specific eNOS KO mice (eNOS^flox/flox^ Hbb-Cre^pos^=RBC eNOS KO) and their respective WT littermate control (eNOS^flox/flox^ Hbb-Cre^neg^) or erythroid-specific eNOS KI mice (eNOS^inv/inv^ Hbb-Cre^pos^=RBC eNOS KI) and their WT littermate control (eNOS^inv/inv^ Hbb-Cre^neg^). In addition, eNOS^flox/flox^ mice were crossed with DelCre mice to create global eNOS KO (gKO). For a structured list of the lines and nomenclature used in the figures and the text please refer to Table 1. For further details on the genetic strategy and selection see the Methods and Figures I and II in the Data Supplement Methods.

**Table 1.**
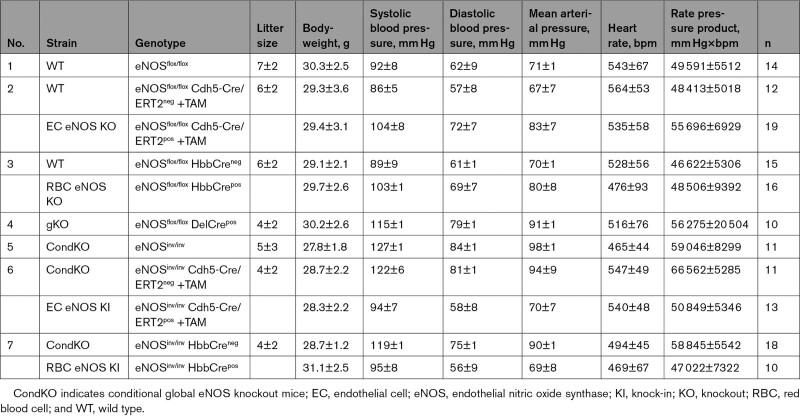
Blood Pressure and Heart Rate in All Strains Investigated

### Analysis of Tissue-Specific Loss-of-Function or Gain-of-Function

The Cre recombinase–dependent genetic locus recombination was determined in targeted and nontargeted tissues by real-time PCR with specific primers and probes designed to recognize the floxed allele and the allele with targeted deletion (Transnetyx). The eNOS and Cre recombinase expression was analyzed by TaqMan real-time reverse transcriptase PCR in EC (CD31^+^ CD45^−^) magnetically isolated from lung homogenates or in erythroid cells (Ter119^+^ CD71^+^ CD45^−^) magnetically isolated from the bone marrow of the mice, and in targeted or nontargeted tissues, as explained in detail in the figures and Data Supplement. The expression of eNOS in RBCs, in RBC membrane preparations (ghosts), and in targeted and nontargeted tissues, was analyzed by immunotransmission electronic microscopy,^21^ Western blot analysis, and quantitative ELISA according to the manufacturer’s protocol (Abcam). The expression of eNOS in RBC lysates was also measured by immunoprecipitation and Western blot analysis according to published procedures.^8^

### Measurements of BP and Systemic Hemodynamics

Invasive assessment of hemodynamic parameters was performed by using a 1.4F Millar pressure-conductance catheter (SPR-839, Millar Instrument) placed into the left ventricle through the right carotid artery according to the closed chest method as described.^22^ Transthoracic echocardiography was performed as previously described.^22^ Left ventricular end-systolic and end-diastolic volumes, left ventricular ejection fraction, fractional shortening, cardiac output, stroke volume, and systemic vascular resistance were calculated. Left ventricular diastolic function was assessed by analysis of the characteristic flow profile of the mitral valve Doppler, which was visualized in an apical 4-chamber view, as described.^22^ For the assessment of hemodynamic responses in awake mice, we used radiotelemetry with a microminiaturized electronic monitor (PA-C10; Data Sciences International) attached to an indwelling aortic catheter. After 3 days of baseline measurements, we measured hemodynamic responses to nitric oxide synthase (NOS) inhibition by the administration of *N*^γ^-nitro-l-arginine methyl ester (L-NAME; 1 mg/mL in drinking water for 3 days; we determined that mice drink 5 mL/d independently of the presence of L-NAME, thus resulting in a dose of 166 mg·kg^–1^·d^–1^); these were followed by hemodynamic responses to increased l-arginine bioavailability achieved by administration of the arginase inhibitor *N*-hydroxy-nor-l-arginine (NorNOHA, 10 mg/kg intraperitoneally for 3 days).

### Measurement of Endothelial Function/Vascular Reactivity Ex Vivo and In Vivo

Thoracic aortas were excised and their functional reactivity analyzed in an organ bath as previously described.^23^ Vascular function in vivo was measured as flow-mediated dilation with a Vevo 2100 with a 30 to 70 MHz linear array microscan transducer (VisualSonics) as described.^22^

### Determination of NO Metabolites in Blood and Tissues

Nitrosated (*S*-nitroso and *N*-nitroso) products (RXNO), and nitrosylheme (NO-heme) were quantified by gas phase chemiluminescence as described.^24^ For nitrite and nitrate analysis, samples were deproteinized with ice-cold methanol (1:1 v/v), cleared by centrifugation, and subjected to analysis by high-performance liquid chromatography using a dedicated nitrite/nitrate analyzer (ENO20, Eicom).^25^

### Statistical Analysis

Sample size was calculated a priori by using G-Power V.3.1 (Heinrich Heine University of Düsseldorf). Statistical analysis was performed with GraphPad Prism 9 for macOS (Version 9.0.2(134)). Unless stated otherwise, the results are reported as means±SD. Normal distribution was tested by the D’Agostino-Pearson test. Comparisons among multiple groups were performed using 1-way and 2-way ANOVA or 2-way repeated measures ANOVA, as appropriate, followed by Tukey or Sidak post hoc analysis, as indicated. Where indicated, unpaired Student *t* test with Welch correction was used to determine if 2 groups of data were significantly different. The Mann-Whitney *U* test was performed when data were not normally distributed. *P*<0.05 was considered statistically significant.

## Results

### Generation of Endothelial and Erythroid-Specific eNOS KO Mice

**Figure 1.**
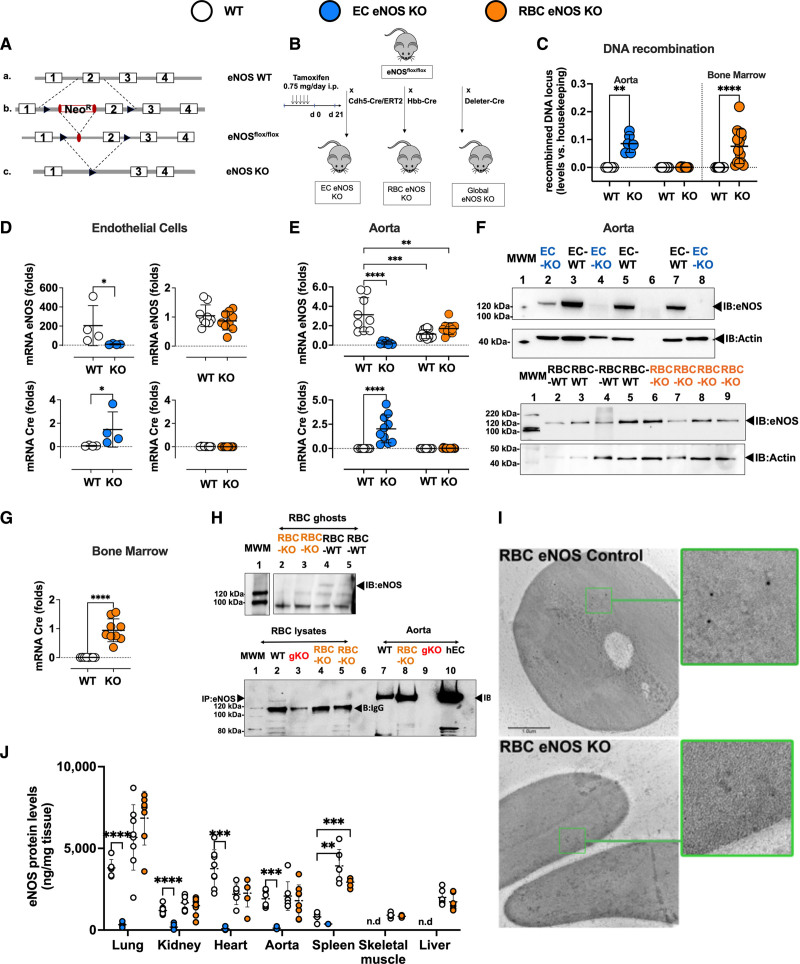
**Generation and characterization of EC eNOS KO or RBC eNOS KO mice. A**, Scheme describing the gene-targeting strategy showing the position of the loxP sequences (black) within the gene-targeting construct before and after exon 2 of Nos3 was used to generate the founder eNOS^flox/flox^ mice. **B**, To generate EC eNOS KO mice and their respective WT control (eNOS^flox/flox^ Cdh5-Cre/ERT2^neg^), the founder eNOS^flox/flox^ mice were crossed with endothelial-specific tamoxifen (TAM)-inducible Cdh5-Cre/ERT2^pos^ mice to obtain eNOS^flox/flox^ Cdh5-Cre/ERT2^pos/neg^ mice; these were treated with TAM for 5 days and analyzed after 21 days. To create RBC eNOS KO mice or global eNOS KO mice and their respective WT littermate controls, the founder eNOS^flox/flox^ was crossed with erythroid-specific (Hbb-Cre^pos^) mice or with Deleter-Cre^pos^ mice (expressing Cre in all tissues). **C**, Real-time PCR analysis shows that tissue-specific DNA recombination occurs in the aorta of EC eNOS KO mice (blue), whereas in RBC eNOS KO mice, DNA recombination occurs in the bone marrow, but not in the aorta (orange). No recombination is observed in WT littermate control mice (white). *t* test **P<0.01, ****P<0.0001 vs respective WT control. **D**, **Top**, Real-time reverse transcriptase PCR analysis shows that endothelial cells (CD31^+^ CD45^−^) extracted from the lung of EC eNOS KO mice (blue) have a significant loss of eNOS expression compared with WT controls. *Mann-Whitney *U* test *P*=0.0286. Instead, the expression of eNOS in lung EC (CD31^+^ CD45^−^) from RBC eNOS KO (orange) is not different from WT control. It is notable that TAM increased the expression of eNOS in lung EC from WT (eNOS^flox/flox^Cdh5-Cre/ERT2^neg^) mice**. Bottom**, Cre recombinase is expressed in lung endothelial cells from EC eNOS KO (blue) but not in endothelial cells from WT (white) or RBC eNOS KO (orange) mice. *Mann-Whitney *U* test *P*=0.0286. **E**, **Top**, Real-time reverse transcriptase PCR analysis shows loss of eNOS expression and Cre recombinase expression in the aorta of EC eNOS KO (blue), but not in WT littermate control mice (white). TAM increased mRNA eNOS expression in the aorta of the WT (eNOS^flox/flox^Cdh5-Cre/ERT2^neg^) mice, but the protein levels are not different from WT mice (see also **I**, ELISA). One-way ANOVA *P*<0.0001; Tukey multiple comparison test **P<0.01,*** *P*<0.001, ****P<0.0001 vs respective WT control. **F**, **Top**, Immunoblot (IB) analysis shows loss of eNOS (135 kDa) expression in the aorta of EC eNOS KO (eNOS^flox/flox^Cdh5-Cre/ERT2^pos^+TAM) mice but not in littermate controls (eNOS^flox/flox^Cdh5-Cre/ERT2^neg^+TAM) after treatment with TAM. Loading control actin (45 kDa). Note: Sample in the first lane shows a residue of eNOS expression in that particular mouse because the knockdown efficiency of this model is ≈90% to 95%. **Bottom**, Immunoblot of aorta of RBC eNOS KO mice and WT littermate controls, showing that eNOS expression is not different in these 2 groups. **G**, Real-time reverse transcriptase PCR analysis shows eNOS expression and Cre recombinase expression in the bone marrow of RBC eNOS KO mice. Cre-recombinase is expressed in the bone marrow of RBC eNOS KO mice, but not in the aorta. Note that, although eNOS deletion was specifically detected in the bone marrow of RBC eNOS KO mice, we determined similar eNOS expression levels in the RBC eNOS KO mice compared with WT littermates; this is attributable to the high abundance of endothelial cells in the bone marrow. ****P<0.0001 Mann-Whitney *U* test vs WT mice. **H**, **Upper**, Immunoblot of membrane preparations of RBCs (ghosts) showing the presence of eNOS in WT RBCs and its absence in RBCs from RBC eNOS KO mice. **Bottom**, Immunoprecipitation (IP) of eNOS (135 KDa) from RBC lysates shows eNOS expression in WT mice and lack of eNOS in global eNOS KO mice (gKO) and RBC eNOS KO mice. The IgG (120 kDa) is seen in the IP samples. **I**, Electron scanning microscopy with immunogold staining of eNOS in WT (eNOS^flox/flox^ HbbCre^pos^) mice (**Upper**) and RBC eNOS KO (**Bottom**). **J**, The quantification of eNOS protein expression in multiple organs by ELISA shows a significant loss of eNOS in multiple tissues of EC eNOS KO mice compared with WT littermate controls. The differences between the WT control groups are not significant, except in the spleen. Data were analyzed according to mixed-effect model with Geisser-Greenhouse correction (variables: strain, tissue); *P*<0.0001; multiple comparisons of eNOS levels among the groups within the same tissue were assessed by a Tukey test **P<0.01; ***P<0.001; ****P<0.000. Lines represent means± SD. EC indicates endothelial cell; eNOS, endothelial nitric oxide synthase; KO, knockout; MWM, molecular weight marker; RBC, red blood cell; and WT, wild type.

To obtain mice specifically lacking eNOS in ECs (EC eNOS KO) or erythroid cells (RBC eNOS KO), we applied the loxP-Cre approach to delete exon 2 of the Nos3 gene. Therefore, an orphan loxP site and an FRT-neo-FRT-loxP resistance cassette were simultaneously inserted into the Nos3 genomic locus to target exon 2 of Nos3 for Cre-mediated excision (Figure 1A). Sequencing confirmed base pair–precise modification of the Nos3 genomic locus and integrity, whereas stable transfection of the construct in embryonic stem cells was assessed by Southern blotting/long-range PCR (see Figure I in the Data Supplement). Two male mice fully derived from A9 embryonic stem cells (hybrid C57/129) were obtained and backcrossed with C57/BL6j mice for at least 10 generations. The eNOS^flox/flox^ mice were crossed with tamoxifen-inducible EC-specific Cre-expressing mice (Cdh5-Cre/ERT2^pos^ mice)^17^; once activated by tamoxifen, the Cre recombinase expressed in eNOS^flox/flox^ Cdh5-Cre/ERT2^pos^ mice removed the floxed segment (Figure 1A), creating EC-specific eNOS KO mice (EC eNOS KO; Figure 1B). In WT (eNOS^flox/flox^ Cdh5-Cre/ERT2^neg^) littermate mice lacking the Cre recombinase, tamoxifen does not lead to genetic recombination.

Tamoxifen-induced Cre expression, DNA recombination, and the resulting genetic deletion of exon 2 of Nos3 in eNOS^flox/flox^ Cdh5-Cre/ERT2^pos^ mice (EC eNOS KO) and the lack thereof in eNOS^flox/flox^ Cdh5-Cre/ERT2^neg^ mice (control) were confirmed by real-time PCR analysis of DNA extracted from vascular tissue (aorta) of the mice (Figure 1C, blue). This resulted in a lack of eNOS expression in lung EC (CD31^+^ CD45^−^; Figure 1D, blue), and in the aorta, as assessed by real-time PCR (Figure 1E, blue) and immunoblot analysis of aortic lysates (Figure 1F). In EC eNOS KO mice, we also found that eNOS expression was nondetectable in highly vascularized tissues such as the lungs, the heart, and the kidney, as assessed by immunoblot analysis (Figure III in the Data Supplement) and quantitative ELISA (Figure 1J).

To create erythroid-specific eNOS KO mice (RBC eNOS KO), eNOS^flox/flox^ mice were crossed with mice constitutively expressing Cre recombinase in erythroid cells under the control of the promoter for the hemoglobin β-chain (Hbb-Cre^pos^^18^; Figure 1B). Cre-dependent deletion of exon 2 was confirmed by real-time PCR in DNA extracted from the bone marrow of the mice (Figure 1C, orange) but was not found in the aorta or other tissues. Cre expression was found in the bone marrow of RBC eNOS KO (Figure 1G, orange), but not in the aorta or other tissues. In RBC eNOS KO we did not detect any eNOS in membrane preparations of RBCs (ghosts) by immunoblotting, in RBC lysates by immunoprecipitation + immunoblotting (Figure 1H), and by scanning electron microscopy and immunogold staining (Figure 1I). On the other hand, in RBC eNOS KO mice, we found that eNOS expression was fully preserved in lung EC (CD31^+^ CD45^−^; Figure 1D, orange), aorta (Figure 1E, orange; Figure 1F, Bottom), and all other tissues of RBC eNOS KO analyzed, including lung, kidney, heart, spleen, skeletal muscle, and liver (Figure 1J; Figure III in the Data Supplement).

### Hematologic Characterization of EC eNOS KO and RBC eNOS KO Mice

Blood counts and rheological parameters remained unchanged in all strains (Table I in the Data Supplement). However, hemoglobin oxygen affinity was significantly higher and oxygen-binding cooperativity significantly lower in RBC eNOS KO mice compared with their WT littermate controls; this difference progressed as pH was lowered within the physiological range (from 7.6 to 7.2; Figure IV in the Data Supplement).

### Vascular Endothelial Function and eNOS Expression Are Compromised in EC eNOS KO but Fully Preserved in RBC eNOS KO Mice

**Figure 2.**
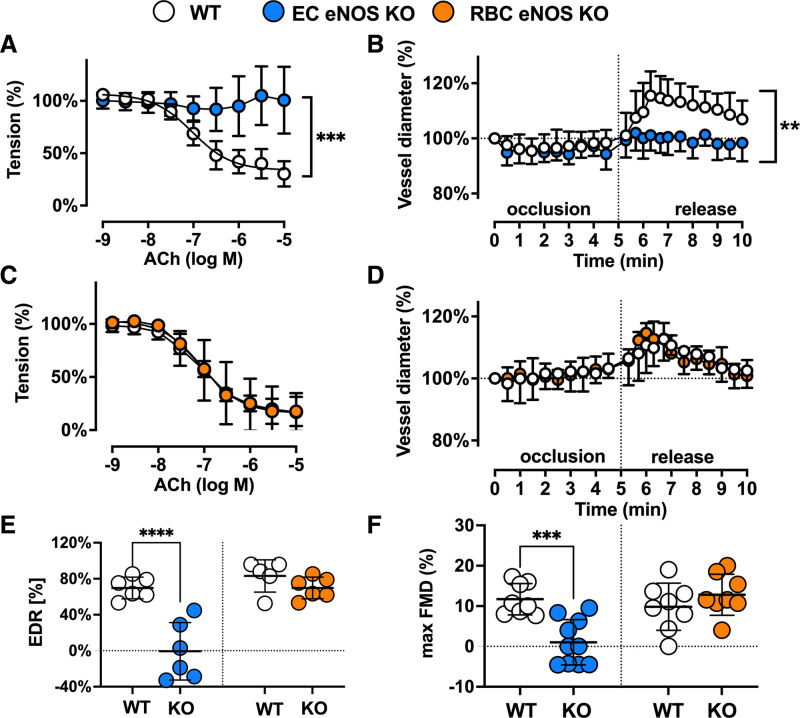
**Vascular endothelial dilator function is lost in EC eNOS KO mice and preserved in RBC eNOS KO mice.** Nitric oxide–dependent vascular endothelial function is fully abolished in EC eNOS KO mice and fully preserved in RBC eNOS KO mice compared with the respective littermate controls. **A**, Preconstricted aortic rings from EC eNOS KO lack acetylcholine (ACh)-induced vasodilation (2-way repeated measurement [RM]-ANOVA *P*<0.0001; Sidak ***P<0.001 vs WT control for concentrations of ACh>10^–6.5^ mol/L (n= 6 per group). **B**, Flow-mediated dilation (FMD) of the iliac artery assessed in vivo by ultrasound is abolished in EC eNOS KO (blue; 2-way RM-ANOVA *P*<0.0001; Sidak **P<0.01 vs WT control [white] for *t*>6.3 minutes; n=10 per group). **C**, In aortic rings from RBC eNOS KO (orange), ACh-induced vasodilation was not different from WT littermates (white). Two-way RM-ANOVA *P*<0.0001. n=5 per group. **D**, FMD of the iliac artery is fully preserved in RBC eNOS KO mice, 2-way RM-ANOVA *P*<0.0001. n=8 per group. **E**, Endothelium-dependent relaxation (EDR) in response to ACh (calculated as the percentage of the maximal ACh response) is significantly impaired in EC eNOS KO and fully preserved in RBC eNOS KO compared with their respective WT controls. One-way ANOVA *P*<0.0001; Tukey test ****P<0.0001. **F**, Maximal FMD (corresponding to the percentage of maximal flow-mediated dilator response) is significantly decreased in EC eNOS KO mice and fully preserved in RBC eNOS KO mice, compared with their respective WT controls. Lines represent means±SD. One-way ANOVA *P*<0.0001; Tukey test ***P<0.001. EC indicates endothelial cell; eNOS, endothelial nitric oxide synthase; KO, knockout; RBC, red blood cell; PCR, polymerase chain reaction; and WT, wild type.

As expected, we found a loss of NO-dependent vascular endothelial function in EC eNOS KO mice, as demonstrated by the lack of acetylcholine (ACh)-induced vasodilation of aortic rings ex vivo (Figure 2A and 2E; Figure V in the Data Supplement), and an abrogated flow-mediated dilation response in the iliac artery as determined by ultrasound in vivo (Figure 2B; Figure VI in the Data Supplement). RBC eNOS KO mice, on the other hand, showed no changes in ACh-mediated vasodilation of aortic rings (Figure 2C and 2E) and a normal flow-mediated dilation (Figure 2D and 2F; Figure VI in the Data Supplement). These results are consistent with fully preserved eNOS expression and activity in the vasculature (Figure 1F and 1J; Figure III in the Data Supplement). Preservation of eNOS activity and function in conductance vessels of RBC eNOS KO mice also indicates that there were no off-target effects of genetic knockdown in vascular tissue. The responses to the constrictor phenylephrine and the NO donor sodium nitroprusside were fully preserved in the aorta of both mouse strains (Figure V in the Data Supplement). In EC eNOS KO, we observed a significant leftward shift in the sodium nitroprusside–mediated response, which indicates an increased sensitivity to NO donors. This response has been observed on inhibition of NOS activity and in different strains of global eNOS KO mice.^26–28^

### Loss of RBC eNOS Leads to Hypertension and an Increase in Systemic Vascular Resistance

**Figure 3.**
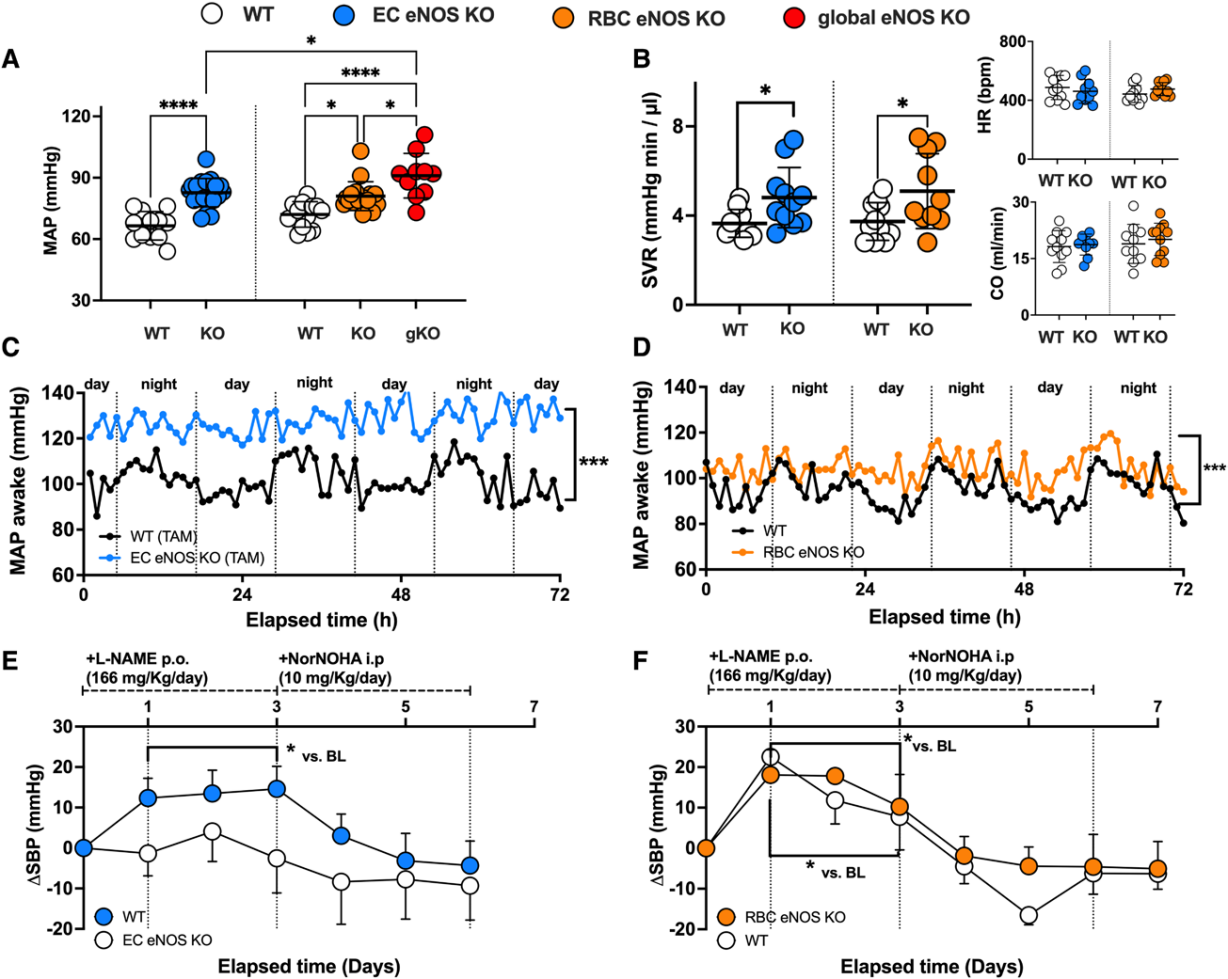
**RBC eNOS and EC eNOS both contribute to blood pressure homeostasis. A**, Invasive measurements of blood pressure (BP) in anesthetized mice show that both EC eNOS KO and RBC eNOS KO mice have increased mean arterial pressure (MAP) compared with their respective WT littermate controls. Global eNOS KO mice (gKO=eNOS^flox/flox^ Deleter Cre) are hypertensive and show significantly higher MAP than EC eNOS KO and RBC eNOS KO mice. One-way ANOVA *P*<0.001; Tukey *P<0.05; ****P<0.0001. **B**, Systemic vascular resistance (SVR) was estimated in a subcohort of mice by measuring MAP and cardiac output (CO) by ultrasound in the same animal (SVR ≈ MAP/CO) and was significantly increased in both EC eNOS KO and RBC eNOS KO mice compared with their respective controls. One-way-ANOVA *P*=0.0149; Welch *t* test *P*<0.05. **Inset Top**, The heart rate (HR) depicted here represents the values measured by cardiac ultrasound. **Inset Bottom**, Cardiac output. See also Table 2 for other cardiac parameters. **C**, Mean of MAP measurements performed in awake EC eNOS KO (n=7) and WT littermates (n=5) by radiotelemetry showing the diurnal and nocturnal variation of blood pressure with highest values during the night. KO (SD = ±10 mm Hg) vs WT (SD = ±8 mm Hg); Welch *t* test *P<0.001. Refer also to Figure VII in the Data Supplement for mean diurnal values. **D**, Mean of MAP measurements performed in awake RBC eNOS KO (n=8) and WT littermates (n=8) by radiotelemetry. KO (SD = ±15 mm Hg) vs WT (SD = ±13 mm Hg); Welch *t* test correction *P<0.001. Refer also to Figure VII in the Data Supplement for mean diurnal values. **E**, Telemetric measurements of changes in systolic BP (SBP) in awake EC eNOS KO mice (n=7) show that NOS inhibition by L-NAME or increase in arginine availability by the administration of the arginase inhibitor NorNOHA did not affect SBP in awake EC eNOS KO mice (blue) compared with WT mice (n=5). Two-way RM ANOVA *P*<0.001; Holm-Sidak *P<0.05 vs baseline. See Figure IV in the Data Supplement for data on diastolic BP and HR. **F**, Radiotelemetric measurements of changes in SBP in a subcohort of awake RBC eNOS KO mice (n=5) show that treatment with L-NAME further increases SBP in RBC eNOS KO and WT littermate (n=3) to the same extent as in the WT controls (although the baseline levels between RBC eNOS KO and WT controls were significantly different, see **C** and **D**, this figure); increase of arginine bioavailability by the administration of NorNOHA rapidly restored BP to the baseline levels. Two-way RM ANOVA *P*<0.001; Holm-Sidak *P<0.05 vs baseline. BL indicates baseline; EC, endothelial cell; eNOS, endothelial nitric oxide synthase; i.p., intraperitoneally; KO, knockout; L-NAME, *N*^γ^-nitro-l-arginine methyl ester; NorNOHA, *N*-hydroxy-nor-l-arginine; p.o., orally; RBC, red blood cell; RM, repeated measures; TAM, tamoxifen; and WT, wild type.

In line with the general knowledge on the role of vascular endothelial eNOS in the regulation of BP, we found that EC eNOS KO mice have significantly higher mean arterial pressure (MAP; Figure 3A, blue) compared with their WT littermates, which is characterized by a difference of 15 to 18 mm Hg in both systolic blood pressure (SBP) and diastolic blood pressure (DBP), whereas heart rate (HR) was similar in both groups (Table 1). RBC eNOS KO mice also had significantly higher MAP than their WT littermates (Figure 3A, orange), characterized by a 14 mm Hg increase in SBP and 8 mm Hg in DBP and no differences in HR (Table 1). In global eNOS KO mice, we measured a 17 to 23 mm Hg increase in SBP and DBP compared with WT mice (Figure 3A, gKO, red). The effect size of eNOS deletion on BP (calculated as Δ% changes in BP versus the respective WT controls) was more prominent in the global eNOS KO (28%–31% increase) and the EC eNOS KO (20%–26% increase) compared with RBC eNOS KOs (13%–16%). Thus, the knockout of eNOS in RBCs modulates vascular tone at the level of smaller resistance arteries, whereas endothelial function in the large conduit arteries is fully preserved.

**Table 2.**
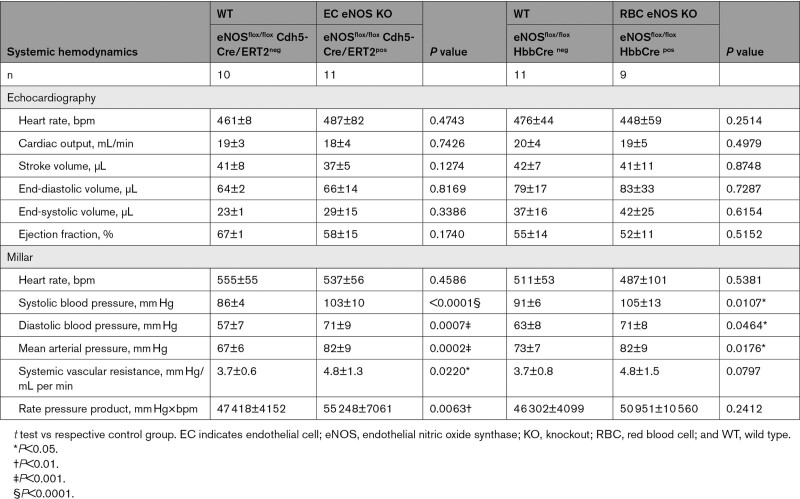
Systemic Hemodynamics as Assessed in a Subcohort of Mice by Measurements of Echocardiography and Millar in the Same Individual

In a subcohort of mice, we characterized systemic hemodynamics by measuring in the same mouse both cardiac function by echocardiography and BP by Millar measurements. We did not observe changes in cardiac output or HR in EC eNOS KO or RBC eNOS KO mice, as assessed by echocardiography (Figure 3B, Inset). HR assessed invasively by a Millar catheter was also not different between groups (Table 2). However, systemic vascular resistance, which was estimated as the ratio between MAP and cardiac output, was higher in both strains than in their respective WT controls (Figure 3B). These results clearly demonstrate that both RBC eNOS and EC eNOS contribute independently to BP homeostasis.

### Hypertension in Awake RBC eNOS KO Mice Can Be Further Increased by NOS Inhibition

Next, we aimed to confirm the BP/HR phenotype in awake mice and analyze the effects of pharmacological regulation of eNOS activity/arginine availability. Therefore, we monitored BP and HR with radiotelemetry in awake mice under untreated conditions for 3 days (Figure 3C and 3D; Figure VII in the Data Supplement). Mice are nocturnal animals, and, as expected, baseline radiotelemetry traces show higher BP levels during the night; it is important to note that diurnal BP levels in awake mice (Figure VII in the Data Supplement) are higher than in anesthetized mice (which were measured during the day), but in awake mice differences between groups remained overall comparable to the one measured in anesthesia (Figure 3C, EC=ΔMAP=20 mm Hg; Figure 3D Δ%=20%; RBC ΔMAP=10 mm Hg; Δ%=10%), confirming hypertension in both EC eNOS KO and RBC eNOS KO mice.

The effects of regulation of eNOS activity on BP in these mice were determined by the administration of a NOS inhibitor (L-NAME) in the drinking water for the next 3 days, which was followed by daily intraperitoneal administration of the arginase inhibitor NorNOHA for the last 3 days of the protocol; NorNOHA was given to the mice to increase the availability of l-arginine to restore eNOS activity after NOS inhibition (Figure 3E and 3F; Figure VIII in the Data Supplement). In EC eNOS KOs, the administration of L-NAME was lethal in 5 of 11 mice tested, but in the mice that survived, it did not significantly affect BP (but decreased HR, see Figure VIII in the Data Supplement) compared with baseline measurements (Figure 3E). In RBC eNOS KO mice, the administration of L-NAME increased SBP by 20 mm Hg (Figure 3F) and decreased HR, whereas DBP was not significantly affected (Figure VII in the Data Supplement). In the WT controls (Figure 3E, white, tamoxifen-treated control; Figure 3D, white, untreated control) L-NAME administration increased SBP by 12 to 20 mm Hg and decreased HR without affecting DBP (Figure VIII in the Data Supplement). Administration of NorNOHA decreased BP by 5 to 10 mm Hg in all mice, thereby restoring BP levels to baseline levels, before L-NAME administration. These data further corroborate that both EC eNOS and RBC eNOS contribute independently to BP regulation in a manner dependent on the availability of the substrate l-arginine.

### Reactivation of eNOS Specifically in RBCs Rescues Global eNOS KO Mice From Hypertension

**Figure 4.**
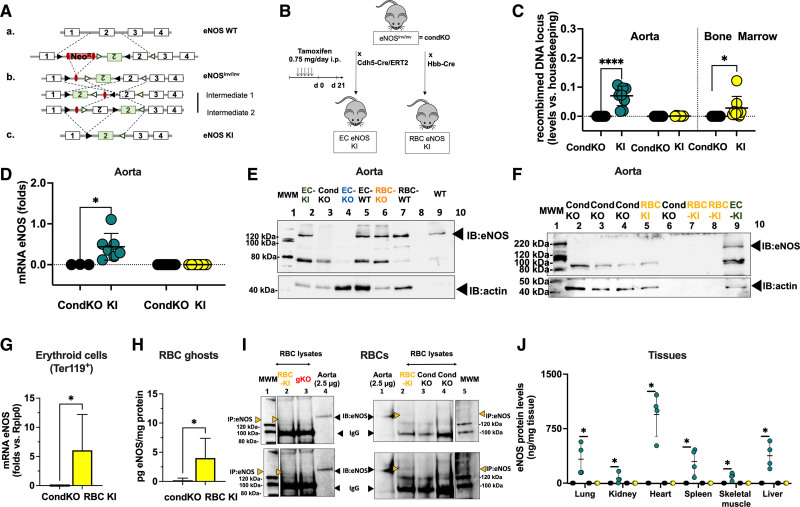
**Reactivation of eNOS expression in either ECs or RBCs rescues global eNOS KO mice from hypertension. A**, Scheme describing the gene-targeting strategy showing the position of a loxP (black) and loxP511 (green) sequences within the gene-targeting construct used to generate eNOS^inv/inv^ mice, which are conditional eNOS KO mice (CondKO). **B**, Schematic representation of the crossing strategy. To create EC eNOS KI mice (green), eNOS^inv/inv^ mice=CondKO mice (black) were crossed with endothelial-specific tamoxifen-inducible Cre mouse (Cdh5-Cre/ERT2^pos^) and treated with tamoxifen for 5 days and analyzed after 21 days. To create RBC eNOS KI mice (yellow), eNOS^inv/inv^ mice=CondKO mice (black) were crossed with erythroid-specific (HbbCre^pos^) mice. **C**, Real-time polymerase chain reaction analysis shows that tissue-specific DNA recombination occurs in the aorta of EC eNOS KI mice (blue), whereas in RBC eNOS KI mice (yellow), DNA recombination occurs in the bone marrow, but not in the aorta. No DNA recombination is observed in CondKO littermate control mice (white). *t* test *P<0.05, ****P<0.0001 vs respective CondKO control. **D**, Real-time reverse transcriptase polymerase chain reaction analysis eNOS expression in the aorta of EC eNOS KI mice (green) but not in CondKO littermate control mice (black). One-way ANOVA *P*<0.0001; Tukey multiple comparisons test *P<0.05 vs respective CondKO control. **E**, Representative immunoblot analysis demonstrating eNOS expression after Cre-dependent reactivation of eNOS expression in aorta EC eNOS KI mice, compared with CondKO, EC eNOS KO, RBC KO, and WT controls. Refer also to **J** (eNOS expression by ELISA) and Figure IX in the Data Supplement for immunoblot analysis from other tissues. **F**, Representative Western blot analysis demonstrating lack of eNOS expression in the aorta of RBC eNOS KI mice and CondKO mice. See Figure IX in the Data Supplement for immunoblot analysis from other tissues. **G**, Real-time reverse transcriptase polymerase chain reaction analysis shows that erythroid cells (Ter119^+^CD71^+^CD45^−^) extracted from the bone marrow of RBC eNOS KI mice (yellow, n=3) express eNOS mRNA compared with CondKO controls, where we could not detect any eNOS mRNA (n=3) *P=0.05 (exact) Mann-Whitney *U* test. **H**, ELISA detection of eNOS expression in RBC membrane preparations (ghosts) from RBC eNOS KI mice (n=4) compared with CondKO controls (n=4), where we could not detect any eNOS protein. *P=0.0286 (exact) Mann-Whitney *U* test. **I**, Low (**Upper**) and high (**Lower**) contrast images representing immunoprecipitation (IP) and immunoblot (IB) analysis of eNOS (135 kDa) from RBC lysates show eNOS expression in RBC eNOS KI mice and lack of eNOS in global eNOS KO mice (gKO) and CondKO mice. The IgG (120 kDa) is seen in the IP samples. **J**, The quantification of eNOS protein expression in multiple organs by ELISA shows a significant eNOS expression in multiple tissues of EC eNOS KI mice, whereas no eNOS was detected in RBC eNOS KI or littermate CondKO mice. Data were analyzed according to the mixed-effect model with Geisser-Greenhouse correction; *P*<0.0001; multiple comparisons of eNOS levels among the groups within the same tissue were assessed by a Tukey test; *P*<0.0001; Tukey *P<0.001. Lines represent means± SD. EC indicates endothelial cell; eNOS, endothelial nitric oxide synthase; KI, knock-in; KO, knockout; MWM, molecular weight marker; RBC, red blood cell; and WT, wild type.

To induce cell type–specific reactivation of eNOS in RBCs or ECs in a global eNOS KO mouse, we created a Cre-inducible eNOS knock-in (KI) gene construct (eNOS^inv/inv^) by inserting an inverted exon 2 and 2 additional Lox511 sites in the loxP-eNOS construct (Figure 4A and Figure VIII in the Data Supplement) This construct allows a Cre-induced reactivation of eNOS in any cell type of interest; therefore, these mice were defined as conditional global eNOS KO mice (CondKO).

As shown in Figure 4B, by breeding eNOS^inv/inv^ with Cdh5-Cre/ERT2^pos^ mice and after tamoxifen treatment, we obtained EC eNOS KI (eNOS^inv/inv^ Cdh5-Cre/ERT2^pos^) with tissue-targeted reactivation of eNOS in ECs only or their littermate global eNOS KO controls (eNOS^inv/inv^ Cdh5-Cre/ERT2^neg^). Tamoxifen-induced DNA recombination and the resulting genetic inversion of exon 2 of Nos3 in eNOS^inv/inv^ Cdh5-Cre/ERT2^pos^ mice (EC eNOS KI), and the lack thereof, in eNOS^inv/inv^ Cdh5-Cre/ERT2^neg^ mice (CondKO control), were all confirmed by real-time PCR analysis of DNA extracted from vascular tissue (aorta) of these mice (Figure 4C, green). This resulted in the reactivation of eNOS expression in the aorta and other tissues, as assessed by real-time reverse transcriptase PCR (Figure 4D), immunoblot (Figure 4E; Figure IX in the Data Supplement), and ELISA of multiple tissues (Figure 4J). In CondKO littermate mice, eNOS expression was nondetectable in any of the tissues analyzed.

In addition, by crossing eNOS^inv/inv^ with Hbb-Cre^pos^ mice, we created RBC eNOS KI (eNOS^inv/inv^ Hbb-Cre^pos^) mice (Figure 4B). These mice are characterized by a targeted eNOS reactivation in bone marrow erythroid cells, as determined by analyzing DNA recombination in the bone marrow (Figure 4C), and eNOS expression in isolated erythroid cells (Ter119^+^ CD71^+^ CD45^−^; Figure 4G), RBC ghosts (Figure 4H), and RBC lysates (Figure 4I), as well, whereas the littermate CondKO mice did not show DNA recombination (Figure 3C, black) or express any eNOS in bone marrow erythroid cells or RBCs (Figure 4G, 4H, and 4I). In all other tissues and cells analyzed, we did not detect any eNOS expression (Figure 4F and 4J; Figure IX in the Data Supplement).

**Figure 5.**
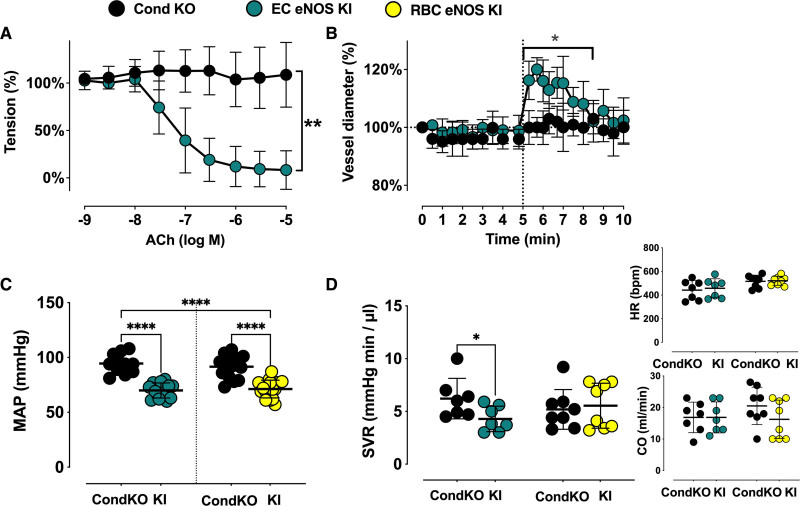
**Reactivation of eNOS expression in either ECs or RBCs rescues global eNOS KO mice from hypertension. A** and **B**, Vascular endothelial dilator function is fully restored in EC eNOS KI. **A**, Preconstricted aortic rings from CondKO mice (black, n=2) lack acetylcholine (ACh)-induced vasodilation, whereas reactivation of eNOS fully restores ACh response in EC eNOS KI (green, n = 3). Two-way repeated-measures ANOVA concentration *P*<0.0001; KI vs KO *P*=0.0461; Sidak **P<0.01 vs CondKO control for concentrations of ACh>10^–7^ mol/L. **B**, Flow-mediated dilation of the iliac artery assessed in vivo by ultrasound is abolished in CondKO mice (black; 2-way repeated-measures ANOVA time *P*=0.0007 KI vs KO *P*=0.329; Fisher least significant difference test **P<0.01 vs CondKO control (black) for *t* >6.3 minutes); and restored in EC eNOS KI mice (green). **C**, Invasive blood pressure analysis shows that reactivation of eNOS expression in ECs in EC eNOS KI mice significantly decreases MAP compared with their littermate conditional eNOS KO mice (CondKO). One-way ANOVA *P*<0.001; Tukey ****P<0.0001. **D**, Systemic vascular resistance (SVR) was estimated in a subcohort of mice by measuring MAP and cardiac output (CO) by ultrasound in the same animal (SVR ≈ MAP/CO). The heart rate depicted here represents the values measured by cardiac ultrasound. SVR is decreased in EC eNOS KI compared with CondKO mice; *t* test Welch *P<0.05. See also Table 3 for other cardiac parameters. CondKO, conditional global eNOS knockout mice; EC, endothelial cell; eNOS, endothelial nitric oxide synthase; KI, knock-in; KO, knockout; MAP, mean arterial pressure; RBC, red blood cell; and WT, wild type.

Of note, in EC eNOS KI mice, reactivation of eNOS specifically in ECs fully restored vascular endothelial dilator function as determined ex vivo (Figure 5A; Figure X in the Data Supplement) and in vivo (Figure 5B; Figure VI in the Data Supplement), and decreased BP by 23 to 28 mm Hg (corresponding to a 25% decrease in MAP; Table 1) and increased systemic vascular resistance (Figure 5D, Table 3) with no significant differences in HR. An important finding was that reactivation of eNOS specifically in erythroid cells fully rescued the global eNOS KO (eNOS^inv/inv^) from hypertension with a significant decrease in BP of 19 to 24 mm Hg (23% decrease in MAP) in the RBC eNOS KI, as compared with their CondKO littermate controls (Table 1 and Table 3). These data provide additional evidence for our finding that RBC eNOS and EC eNOS contribute independently to BP homeostasis.

**Table 3.**
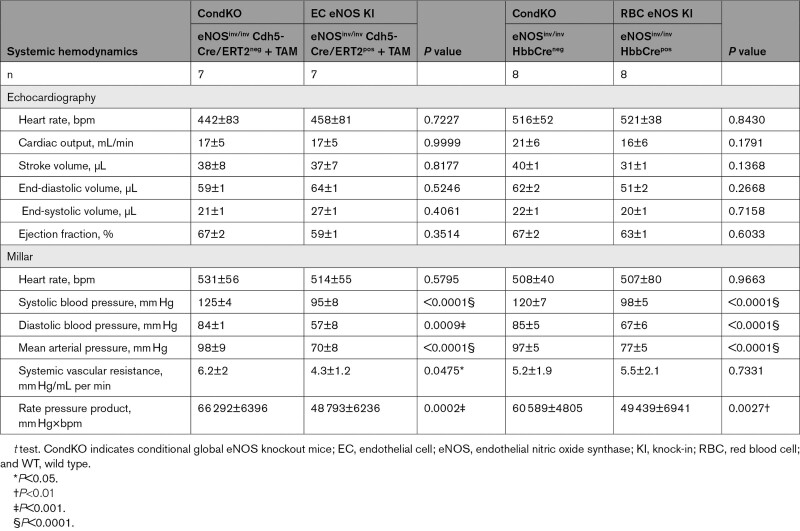
Hemodynamics of EC eNOS KI and RBC eNOS KI mice and Their respective Controls

### Circulating Nitrite Is Decreased in Both EC eNOS KO and RBC eNOS KO and the Levels of NO-Heme Are Changed in RBC eNOS KO/KI Mice

**Figure 6.**
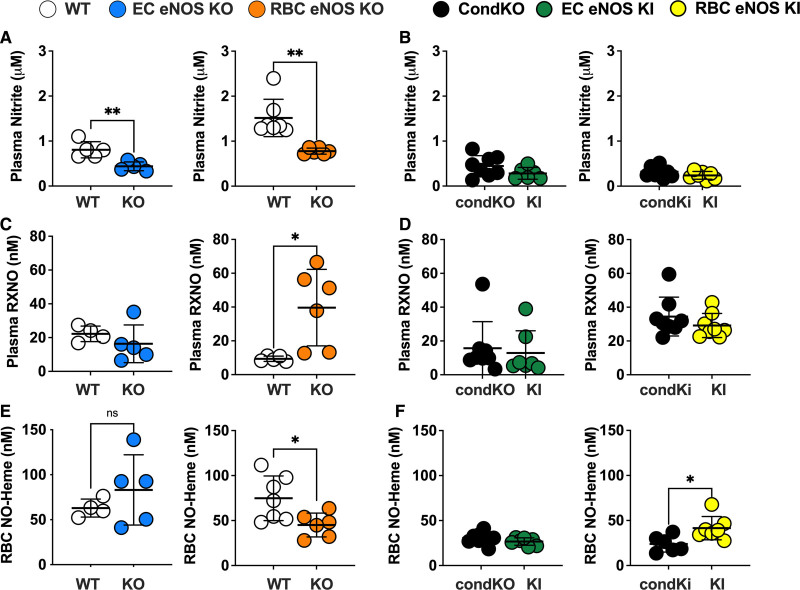
**RBC eNOS and EC eNOS both contribute to plasma nitrite, but red cell eNOS is the major determinant of circulating NO-heme. A**, A significant decrease in plasma nitrite is observed in both EC eNOS KO (blue) and RBC eNOS KO (orange), compared with their respective WT controls (white). Welch *t* test *P<0.05; **P<0.01. **B**, Plasma nitrite in EC eNOS KI (green) or RBC eNOS KI (yellow) is not different from CondKO mice (black), but CondKO mice show lower plasma nitrite levels than WT mice (white; see also Table 4). **C**, The levels of nitrosospecies (RXNO=RSNO+RNNO) were unchanged in EC eNOS KO (blue) and significantly higher in the plasma of RBC eNOS KO (orange) mice, compared with their respective WT control (white). Welch *t* test *P<0.05. **D**, The levels of RXNO in plasma of EC eNOS KI, RBC eNOS KI, and CondKO mice are comparable. **E**, NO-heme concentrations in RBCs were unchanged in EC eNOS KO mice and decreased in RBC eNOS KO mice. Welch *t* test *P<0.05. **F**, Accordingly, concentrations of NO-heme were unchanged in EC eNOS KI mice (green) but higher in RBC eNOS KI mice (yellow) compared with CondKO mice (black), indicating that RBC eNOS is the major determinant of circulating NO-heme. Welch *t* test *P<0.05. CondKO, conditional global eNOS knockout mice; EC, endothelial cell; eNOS, endothelial nitric oxide synthase; KI, knock-in; KO, knockout; NO, nitric oxide; RBC, red blood cell; RXNO, nitrosospecies (nitrosothiols+nitrosamines); and WT, wild type.

Next, we aimed to analyze the impact of cell-specific eNOS deletion on the concentrations of NO metabolites in blood and tissues of all KO and KI lines. In EC eNOS KO mice, we found a decrease in circulating nitrite and nitrate levels in plasma (Figure 6A and 6B; Table 4), without significant changes of these 2 metabolites in RBCs or aorta (Table 4). We also found a decrease in total NO species (ie, the sum of all NO species assessed in a specific compartment) in plasma, aorta, and lung of EC eNOS KO mice, compared with their WT littermates (Table 4); however, decreases in the aorta and plasma were not statistically significant. In the liver, on the other hand, we found an increase in nitrate, contributing to an overall increase in total NO species (Table 4). This is consistent with the observation that the liver may act as a reservoir of nitrate.^29^ Similar changes as observed in EC eNOS KO were observed in global eNOS KO mice by us and others before.^5,6,24,30^

**Table 4.**
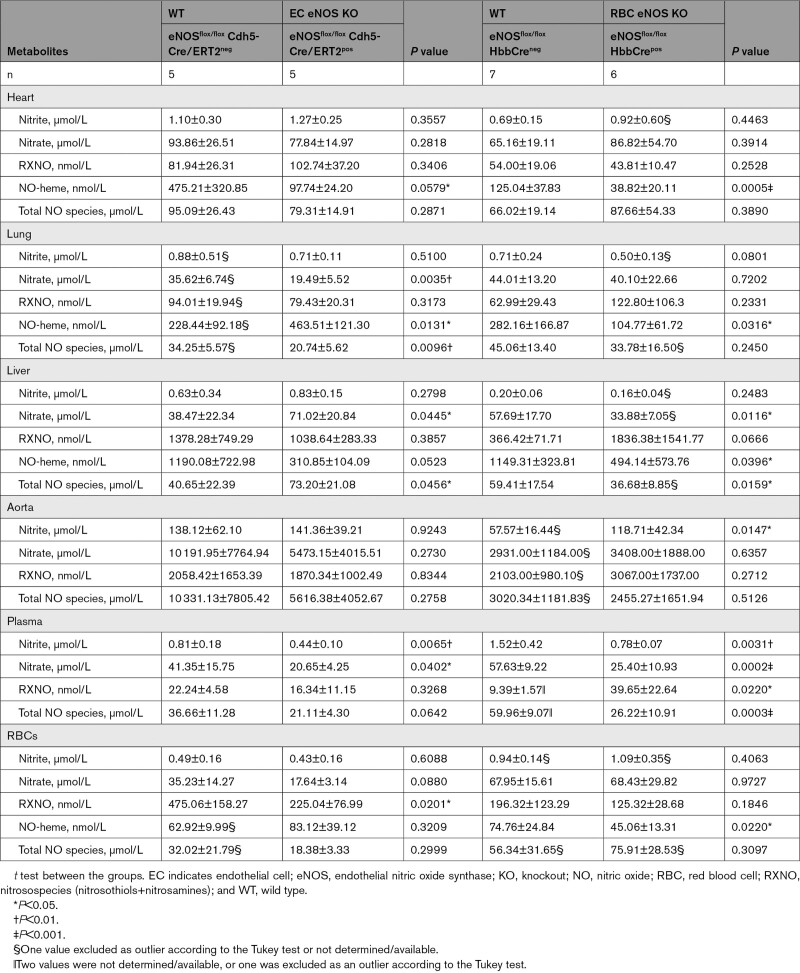
NO Metabolites in Blood and Organs of EC eNOS KO, RBC eNOS KO, and Corresponding WT Littermate Controls

RBC eNOS KOs showed alterations in nitrite and nitrate concentrations in plasma and aorta. Specifically, we found a significant decrease in nitrite and nitrate in plasma (Figure 6A, Table 4), together with an increase in nitroso species (Figure 6C) compared with WT control mice. In the RBC eNOS KO mice, we observed a significant decrease in total NO species in plasma (Table 4), but no other significant changes in any other tissues, except for an increase in nitrite in the aorta compared with WT controls (Table 4).

CondKO mice showed an overall decrease in NO metabolites in plasma and tissues compared with WT mice (compare Table 4, WT with Table 5, Cond KO), with an almost 50% decrease in circulating nitrite levels (Figure 6B, black). However, nitrite levels in the plasma of EC eNOS KI or RBC eNOS KI were not different from their littermate global eNOS KO mice (Figure 6B, green and yellow triangles). These data show that plasma nitrite levels depend on the presence of eNOS in both the ECs and the RBCs and decrease in the global absence of eNOS, but the reintroduction of eNOS in ECs or RBCs does not restore nitrite levels (as it does with BP; see also Figure XII in the Data Supplement).

**Table 5.**
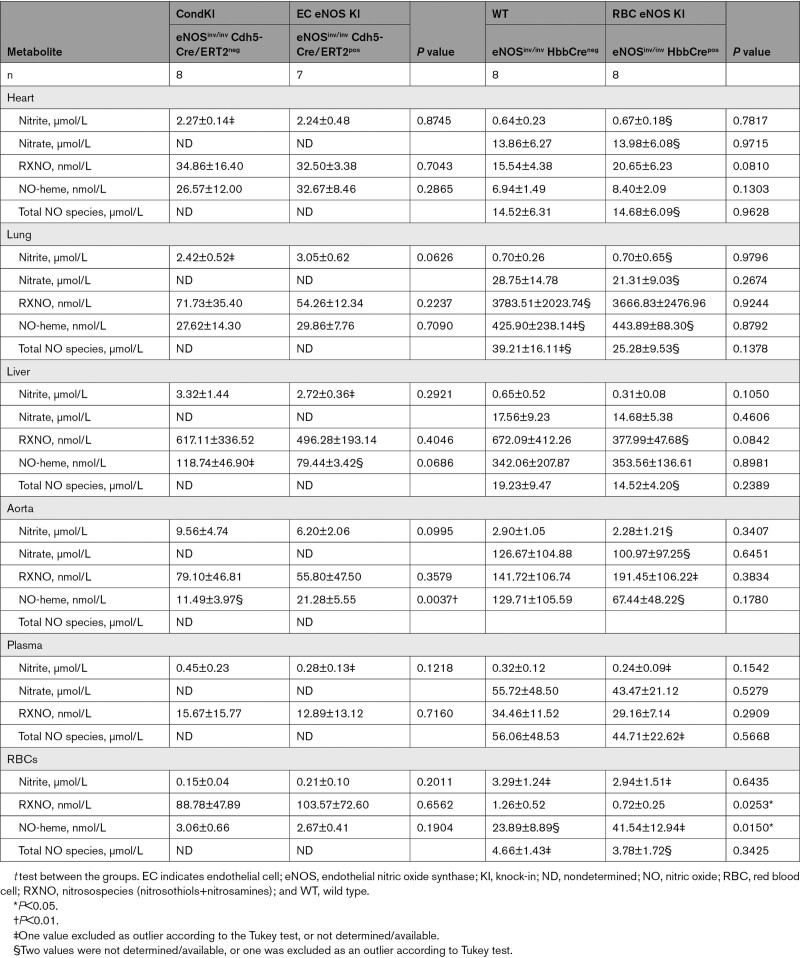
NO Metabolites in Blood and Organs of EC eNOS KI, RBC eNOS KI, and Corresponding Conditional Global eNOS Knockout Mouse Littermate Controls

The levels of bound NO (measured as NO-heme concentrations) were fully preserved in RBCs from EC eNOS KO mice (Figure 6C, blue) but significantly lower in RBCs from RBC eNOS KOs (Figure 6C, Right, orange) and CondKO mice (Figure 6C, black). Accordingly, levels of NO-heme were not altered in RBCs from EC eNOS KI mice (Figure 4C, green), but elevated in RBCs from RBC eNOS KI mice (Figure 4C, yellow), compared with the respective CondKO littermates. These data show that the levels of bound NO in RBCs depend on the presence/absence of eNOS in RBCs.

## Discussion

In this work, we were able to determine the independent contribution of vascular ECs and RBCs to NO metabolism, vascular function, and BP homeostasis by creating and comparing tissue-specific eNOS KO and eNOS KI mice in ECs or RBCs. As expected, we find that eNOS in the vascular wall is the main determinant of endothelial function in conduit and resistance arteries. It is surprising that eNOS in RBCs effectively contributes to BP homeostasis, tissue perfusion, and systemic circulatory NO bioavailability independently of vascular eNOS.

Previous work relied solely on the use of global eNOS KOs or pharmacological inhibition with drugs targeting all 3 NOS isoforms. By creating mice lacking eNOS only in ECs, we can now ultimately confirm the suggested central role of these cells in eNOS-mediated control of cardiovascular function as originally proposed when endothelium-derived relaxing factor was discovered.^31^ The fact that RBC eNOS KO mice were also hypertensive was definitely more surprising, although a role for blood cell eNOS had been suggested in 1 study using chimeric mice obtained by transplanting bone marrow from eNOS KO mice into irradiated WT mice, and vice versa.^9^ The functional significance of eNOS in RBCs has been a matter of intense debate for some time.^10,11,14,32,33^

We previously demonstrated that eNOS is present and active in RBCs.^8,34^ The data presented here show that genetic deletion of eNOS specifically in erythroid cells leads to significant increases in SBP and DBP. These changes were not accompanied by changes in HR and cardiac output, but by increases in systemic vascular resistance. In RBC eNOS KO mice, eNOS activity and function at the level of conduit and proximal resistance arteries were fully preserved. In line with these observations, the administration of a NOS inhibitor increased BP further in RBC eNOS KO mice. Moreover, the administration of an arginase inhibitor to restore arginine availability counteracted the effect of NOS inhibition and quickly restored baseline conditions, showing that, in RBC eNOS KO mice, both regulatory components are active and can be modulated by pharmacological intervention. Last, when eNOS was reintroduced into either ECs or RBCs of conditional global eNOS KO mice, it successfully rescued the hypertensive phenotype in both cases. These data strongly affirm the role of eNOS in both cell types as being essential to BP regulation.

BP homeostasis is primarily regulated at the level of distal conduit, proximal and distal resistance, and precapillary sphincter arteries. Our findings underscore unequivocally that an l-arginase-eNOS signaling cascade is effectively expressed in ECs and RBCs and, by comparing the phenotype of the 4 mice lines presented here, it becomes clear that, depending on its localization, this pathway exerts distinct and complementary physiological effects on NO metabolism and BP control. This may be because of the specific (sub)compartmentalization of eNOS within the vessel wall and its cellular components,^35–38^ the unique anatomy and function of the vascular tree^39^ and its relationship with the circulating blood cells,^14^ and the different half-life and distribution kinetics of the NO metabolites within the blood and the tissues.^9,16,24,40–42^ The functional differences of the pathway in RBCs or the vasculature may become prominent under specific pathophysiological conditions such as endothelial dysfunction or blood disorders.

It is well known that eNOS protein expression and activity in RBCs are both significantly lower than in the endothelium.^8^ However, according to a recent estimate of the total number of cells in the human body, RBCs were the largest contributor to the overall number.^43^ Thus, total RBC numbers may compensate for lower eNOS activity per cell. Therefore, the high density of RBCs and extensive surface area might provide sufficient eNOS-derived NO signaling/export of NO bioactivity to contribute to blood flow and BP regulation where arterial vessel size decreases.

Moreover, it has been shown previously that endothelial eNOS expression declines from proximal to distal sites of the arterial and coronary circulation.^39^ Conversely, RBCs carry eNOS uniformly along the vascular tree, and their contact with the vessel wall becomes more intimate as they travel from proximal to distal sites of the arterial circulation. The smaller distance to the endothelial surface may facilitate the effectiveness of the eNOS-dependent export of NO bioactivity from RBCs. This may explain in part the distinct size effects of induction and rescue of arterial hypertension using our cell-specific eNOS KO/KI mice in the absence and presence of NOS/Arg inhibitors (L-NAME and NorNOHA) and, in particular, the apparent lack of effect of L-NAME in EC eNOS KO mice (which was accompanied by increased mortality in this group only). This points to the existence of 2 fundamental and complementary eNOS-dependent pathways controlling BP hemostasis.

Further regulatory components are the nature, distribution, and kinetics of the several NO metabolites found in plasma, RBCs, and vascular tissues.^24,40,44^ Under normal physiological conditions, the main enzymatic source of NO in the body is thought to be the endothelial eNOS,^6,41^ with lesser contributions of the neuronal NOS and the inducible NOS (in monocytes/macrophages). It was suggested that eNOS-derived NO may exert endocrine effects that are thought to be mediated mainly by nitrite and other semistable circulating metabolites; these include nitrate, nitroso compounds, and possibly nitro fatty acids,^24,40,44^ which can be formed in blood and tissues by several reactions. Based on ex vivo experiments, RBC eNOS is proposed to control the release of NO metabolites from RBCs.^9,10,34,45^

The data presented here show that both EC eNOS and RBC eNOS contribute to the levels of systemic NO metabolites, albeit in different ways. In EC eNOS KO mice, the overall amount of NO metabolites was decreased mainly in plasma, lung, and heart, whereas in the RBC eNOS KO mice clear changes are observed only in the blood, which further supports the notion of differential regulation of the circulating NO pool and NO bioactivity affecting vascular tone as generated from either EC or RBC eNOS. The levels of RXNO in plasma or RBCs show some variability (which was not observed in the other groups) and show no correlation with the presence/absence of eNOS in these models.

When looking specifically at the levels of NO-heme in RBCs, which has been proposed to be a good indicator of EC eNOS-derived NO and systemic NO bioavailability,^46^ we surprisingly found that RBC eNOS rather than EC eNOS was the major contributor, as clearly demonstrated by comparing the levels of NO-heme in the 4 mouse lines analyzed. This indeed suggests that eNOS in the RBCs is functional and is 1 important source of NO bound in RBCs.

Signaling by eNOS in both erythroid and endothelial bone marrow cells has been proposed to control the differentiation of erythroid cells and hematopoiesis.^47^ We did not observe any changes in the hematologic phenotype in our mice: blood count, hematocrit, and blood RBC deformability were not significantly different in RBC eNOS KO mice or EC eNOS KO mice, limiting the role of rheological changes in BP phenotype in those mice. However, oxygen affinity was significantly higher and oxygen-binding cooperativity was significantly lower in the RBC eNOS KO mice; this difference progressed when pH was lowered within the physiological range (from 7.6 to 7.2), indicating a role of RBC eNOS in the Bohr effect, which had earlier been proposed to occur by *S*-nitrosation of Band 3/Anion exchanger 1.^48^

The specificity of gene-targeting deletion or reactivation of eNOS in 1 specific cell type relies on the specificity of the promoter regulating the Cre recombinase. Both the cadherin-5 promoter and the promoter for the hemoglobin β-chain were previously characterized as being highly specific for the endothelial^17^ and erythroid^18^ compartment, respectively. It is important to point out that some chimerism was observed for the expression of Cre recombinase in megakaryocytes,^18^ the precursors of platelets. Although the presence and role of eNOS in megakaryocytes and platelets is still highly controversial and its role in BP regulation unknown, it cannot be excluded a priori. Nevertheless, the efficiency and specificity of EC or RBC targeting were verified here by multiple independent techniques ranging from detection of DNA recombination to cell- and tissue-specific expression of eNOS and Cre recombinase in isolated cells and tissues.

How then does RBC eNOS contribute to BP regulation? At the moment, this question can only be speculated on. Numerous studies suggest that RBCs are capable of exporting NO bioactivity under hypoxic conditions,^45,48,49^ although the identity of the species released responsible for carrying and exerting NO-like bioactivity is yet to be determined.

The finding of decreased NO-heme levels in RBC eNOS KO mice and increased NO-heme levels in RBC eNOS KI mice is very interesting. The main heme protein in RBCs is hemoglobin (Hb) and nitrosyl-Hb (heme iron-bound NO, Fe^2+^-NO) is the central precursor in 2 hypotheses related to the export of NO bioactivity from RBCs; the one involving nitrosation of Cys-β93 in Hb^50^ and the other one involving deoxyhemoglobin-mediated reduction of nitrite to NO.^49^ The key point of the controversy is how nitrosyl-Hb is formed and whether nitrosyl-Hb is simply an inert biomarker for NO levels in the RBCs, or rather it is a reservoir of NO, if it could be released.^51^

During NO inhalation, NO-heme forms in the pulmonary vasculature and is rapidly metabolized from artery to vein, suggesting its ability to undergo an oxidative denitrosylation along the vascular tree (ie, its conversion into Fe^2+^-NO to Fe^3+^ and release of NO). The proposed mechanism consists of concerted reactions of oxyHb and nitrite generating oxidative intermediates (H_2_O_2_ and NO_2_) that convert Fe^2+^-NO to Fe^3+^+NO and N_2_O_3_ that can escape Hb autocapture.^52^ As RBCs deoxygenate, nitrosyl-Hb and nitrite participate in reactions that generate NO, NO_2_, and N_2_O_3_. A further mechanism for bioactivity related to nitrosyl-Hb is the recently proposed release of the entire NO-heme complex to function as a signaling entity,^53^ but its functional significance is still under investigation.

Nevertheless, here, we presented genetic evidence linking the presence of eNOS in RBCs with the levels of NO bound to RBCs. If one considers that eNOS-derived NO formation from l-arginine requires O_2_ and the formation of nitrosyl-Hb may result from RBC eNOS activity, these data indicate that in RBCs an interesting interplay of eNOS-dependent NO formation, formation of nitrosyl-Hb, and export of NO bioactivity related to Po_2_ may occur.

Another possibility is that the RBC is a net exporter of nitrite, which has been shown to act as a vasodilator and regulator of vascular tone independently of EC eNOS^49^ through mechanisms including myoglobin- or xanthine oxidase–mediated nitrite reduction in the vessel wall.^44,54^ Nitrite and iron-nitrosyl-Hb likely relate inversely to BP changes, as suggested by independent groups^7,49,55,56^ and confirmed with recent translational human studies.^57,58^

The data presented here show that the lack of eNOS in the RBCs and concomitant decrease in NO levels in RBCs (in form of NO-heme) and plasma correspond to an increase in BP, without affecting the activity or function of eNOS in the endothelium. They could indicate a paracrine role of nitrite/NO metabolites in the regulation of BP^49^ and demonstrate a central role of eNOS-dependent signaling in RBCs. Further confirmation of this hypothesis is the observation that conditional global eNOS KO mice show a ≈50% decrease of circulating nitrite compared with the WT mice. However, eNOS reactivation in either compartment did not restore circulating nitrite levels in these models, although BP was comparable to that in WT mice. These findings suggest that in a global eNOS KO the mechanism governing the regulation of plasma nitrite is only in part dependent on vascular/RBC eNOS–derived NO production.

A common uncertainty when trying to explain RBC export of NO bioactivity relates to where the NO is originating from. Most authors would suggest that the endothelium is the major source and that NO is first picked up by the RBCs.^1,46^ Despite being present at very low levels, the data presented here actually show that RBC eNOS plays a role both in regulating circulating levels of nitrite/nitrate and NO-heme levels in the RBCs, and BP homeostasis, as well. It is most important that we could demonstrate that NO bioactivity exported from RBCs is effective in the regulation of vascular function at the level of resistance arteries, although it does not affect conduit arteries, because RBC eNOS KO mice have increased MAP but preserved function of conduit arteries.

These distinct effects of eNOS on arterial macro- and microcirculation are in line with the concept that multiple layers of compartmentalization are in place to regulate eNOS-derived NO bioactivity, as presented by independent authors.^35–39^ In the bloodstream, RBCs are located closer to the endothelium downstream in the circulation, whereas, in the large elastic conduit arteries, RBCs are aligned mainly in the central bloodstream far away from the endothelial lining, thus limiting NO scavenging upstream and allowing the release of NO bioactivity downstream.^14,16^

In the vessel wall, eNOS is found both at the luminal side of ECs (apical EC) and the basal side of ECs. Endothelial NO is released abluminally from ECs toward the vascular smooth muscle, and, to a lesser extent, as a spillover into the blood at its luminal site. In addition, specifically in resistance arteries, NO goes through myoendothelial junctions, and its transfer from ECs to smooth muscle cells depends on the redox state of hemoglobin alpha.^37,38^ Accordingly, the contribution of ECs or RBCs to the circulating NO pool and regulation of vascular tone may differ substantially along the arterial circulation, and RBC-mediated release of NO bioactivity and its vasodilatory potential may critically depend on the diameter of the respective arterial segment.

A further aspect that contributes to the complexity of the interplay between ECs and RBCs is the nature and stability of NO metabolites carrying NO bioactivity, their kinetics, and their longitudinal and transversal gradients within arterial segments that may affect their vasodilatory bioactivity. The complexity of these 3 components controlling arterial NO bioactivity (the blood, the vessels, and the NO metabolites) underscores the need for biochemical and functional assessment in vivo of cell-specific eNOS activity from either ECs or RBCs, which, according to our data, may represent the 2 major cell types involved in the regulation of tissue perfusion.

In summary, we created a series of novel mouse models for tissue-specific gene targeting of eNOS. The comparison of mice lacking eNOS specifically in the endothelium or erythroid cells demonstrates that eNOS plays independent roles in these 2 cellular compartments. We present here, for the first time, compelling evidence demonstrating that RBC eNOS directly contributes to systemic NO bioavailability and BP homeostasis, independently of vascular endothelial eNOS. The data provided herein, and the availability of the mice models, will allow for a better understanding of the specific role that RBC eNOS plays in normal physiology and in disease conditions, as well. These include coronary artery disease and chronic kidney disease, where RBC eNOS is decreased,^8,59,60^ hematologic diseases and hemoglobinopathies, which are characterized by a systemic decrease in NO bioavailability and defects in eNOS signaling, like in sickle cell disease.^61^ Moreover, our data and models may also help understanding how RBC eNOS signaling affects RBC function, NO scavenging, NO/sulfide cross-talk, and oxygen transport^62^ and may enable us to refine the criteria for blood banking and transfusion.^63^

## Acknowledgments

The authors thank S. Becher, E. Bruns, J. Hocks, M. Mikus-Lelinska, W. Lückstadt, C. Nihlén, A. Olsson, S. Thasian-Sivarajah, and N. Thomas for their skillful assistance in the experiments. The authors are indebted to S. Münch, C. Gourgoula, and P. Benten (Zentrale Einrichtung für Tierforschung und wissenschaftliche Tierschutzaufgaben [ZETT]; Heinrich-Heine-Universität Düsseldorf [HHU]) for the help with the breeding and maintenance of the mice lines, and to Dr M. Hoffmann and all the colleagues of the Research Laboratory of the Clinic for Urology (Universitätsklinikum Düsseldorf [UKD]) for the use of their gel documentation system ChemiDoc (BioRad) and the flow cytometer MACS quant (Miltenyi Biotech). Moreover, they thank Dr M. Carlström (Department of Physiology and Pharmacology, Karolinska Institutet) for the inspiring discussion about the role of NO metabolites in hemodynamics, Drs A. Heinen and A. Gödecke (Institute of Physiology, HHU) for the discussions about the determination of systemic vascular resistance, the use of their Vevo. and the invaluable and expert help.

## Sources of Funding

This work was supported by the German Research Council (DFG CO 1305/2-1 to Dr Cortese-Krott; DFG SFB 1116 TP B06 to Drs Cortese-Krott and Kelm; TP B10 to Dr Grandoch; the IRTG1902 TP9 to Drs Cortese-Krott and Kelm; TP10 to Dr Stegbauer; TP12 to Dr Grandoch) and by independent research grants of the Forschungskommission, Medical Faculty, Heinrich-Heine University Düsseldorf (to Dr Cortese-Krott; to Dr Suvorava; to Drs Cortese-Krott and Stegbauer). Dr Leo is a scholar of the IRTG1902. Mr Li, Mrs LoBue, Drs Hutzler, and Barbarino are scholars of the SPP1710. Dr Feelisch acknowledges funding from the Medical Research Council and the Wellcome Trust. Dr Isakson was supported by National Institutes of Health HL088554. Dr Cortese-Krott is a visiting professor and Wenner-Gren research fellow in the Department of Physiology and Pharmacology, Karolinska Institutet.

## Disclosures

None.

## Supplemental Materials

Expanded Methods

Data Supplement Table I

Data Supplement Figures I–XII

## Supplementary Material


