# Supplementary material for: Red Blood Cell and Endothelial eNOS Independently Regulate Circulating Nitric Oxide Metabolites and Blood Pressure
Source: Circulation. 2021 Jul 7;144(11):870–89. doi: 10.1161/CIRCULATIONAHA.120.049606 (PMC8529898; doi:10.1161/CIRCULATIONAHA.120.049606)
Supplement: Supplementary file 1 [file cir-144-870-s001.pdf]

SUPPLEMENTAL MATERIAL

# Red blood cell and endothelial eNOS independently regulate circulating nitric oxide metabolites and blood pressure

Francesca Leo, MSc[1,*]; Tatsiana Suvorava, PhD[1,10*]; Sophia K. Heuser, MSc[1]; Junjie Li, MSc[1]; Anthea LoBue, MSc[1]; Frederik Barbarino, PhD[1]; Eugenia Piragine, PhD[1,2]; Rebekka Schneckmann, PhD[3]; Beate Hutzler, PhD[1]; Miranda E. Good, PhD[4,5]; Bernadette O. Fernandez, PhD[6]; Lukas Vornholz, PhD[1]; Stephen Rogers, PhD[7]; Allan Doctor, MD[7]; Maria Grandoch, MD[3]; Johannes Stegbauer, MD[8]; Eddie Weitzberg, MD, PhD[9]; Martin Feelisch, PhD[6]; Jon O. Lundberg, MD; PhD[9], Brant E. Isakson, PhD[4]; Malte Kelm, MD[10,11]; Miriam M. Cortese-Krott, PhD[1,9, 10].

*Contributed equally.

*[1]Myocardial Infarction Research Laboratory, Department of Cardiology, Pulmonology, and Angiology, Medical Faculty, Heinrich-Heine-University, Düsseldorf, Germany; [2]Department of Pharmacy, University of Pisa, Pisa, Italy; [3]Department of Pharmacology and Clinical Pharmacology, Medical Faculty, Heinrich-Heine-University, Düsseldorf, Germany; [4]Robert M. Berne Cardiovascular Research Center, Department of Molecular Physiology and Biophysics, University of Virginia School of Medicine, Charlottesville, VA, USA; [5]Molecular Cardiology Research Institute, Tufts Medical Center, Boston, MA, USA; [6]Clinical & Experimental Sciences, Faculty of Medicine, University of Southampton, Southampton, United Kingdom; [7]Department of Pediatrics, Center for Blood Oxygen Transport and Hemostasis, University of Maryland School of Medicine, HSF III, Baltimore, MD, USA; [8]Department of Nephrology, Medical Faculty, Heinrich-Heine-University, Düsseldorf, Germany; [9]Department of Physiology and Pharmacology, Karolinska Institute, Stockholm, Sweden; [10]Department of Cardiology Pneumology and Angiology, Medical Faculty, Heinrich-Heine-University, Düsseldorf, Germany; [11]CARID, Cardiovascular Research Institute Düsseldorf, Medical Faculty, Heinrich-Heine-University, Düsseldorf, Germany.*

**Short title (max 50):** Red cell eNOS and blood pressure control.

**Correspondence to:**

Miriam M. Cortese-Krott, PhD
Myocardial Infarction Research Laboratory,
Department of Cardiology, Pulmonology, and Vascular Medicine,
Medical Faculty, Heinrich-Heine-University of Düsseldorf
Postfach 128, Universitätsstrasse 1, 40225 Düsseldorf, Germany
E-Mail: Miriam.cortese@hhu.de
Tel.: +49 (0) 211 81 15115

## Expanded Methods

The data that support the findings of this study are available from the corresponding author on reasonable request.

### Materials

Unless otherwise specified, chemicals were purchased from Sigma-Aldrich Co. LLC. (Deisenhofen, Germany). Materials for western blotting were purchased from Life Technologies (Invitrogen, Darmstadt, Germany).

### Animals

All experiments were approved by the LANUV according to the European Convention for the Protection of Vertebrate Animals used for Experimental and other Scientific Purposes (Council of Europe Treaty Series No. 123). Animal care was provided following the institutional guidelines. Mice expressing Cre recombinase in erythroid cells under the control of the promoter of the hemoglobin beta chain (C57BL/6-Tg(Hbb-Cre)12Kpe/J; MGI: J:89725)[18] were obtained by Jackson Laboratory (JAX stock #008314) and crossed for more than 10 generations with C57BL/6J. Tamoxifen-inducible endothelial-specific Cre mice (Tg(Cdh5-Cre/ERT2)1Rha; MGI:3848982)[17] were kindly provided by Prof. Dr. E. Lammert, (Heinrich-Heine-University of Düsseldorf, Düsseldorf, Germany). DeleterCre (C57Bl/6.C-Tg(CMVCre)1Cgn/J)[19] mice expressing Cre in all tissues were kindly provided by Prof. Claus Pfeffer (Heinrich Heine University of Düsseldorf). Experimental planning and execution followed the ARRIVE recommendations[20]. For experiments, 2-6 months old male mice up to 30 g were used. Mice of the same genotype and age were randomly assigned to experimental groups. Evaluation of data obtained by ultrasound was carried out by a blinded researcher.

### Generation of EC/RBC eNOS KO and EC/RBC eNOS KI mice.

Two independent founder lines carrying a floxed eNOS (eNOS$^{flox/flox}$) or a Cre-inducible eNOS knock-in (KI) gene construct (eNOS$^{inv/inv}$) were created, allowing targeted removal or reactivation of eNOS in either EC or RBCs, or in all cells. To generate eNOS$^{flox/flox}$ mice we designed a loxP eNOS targeting construct by simultaneously inserting an orphan loxP site and a FRT-neo-FRT-loxP resistance cassette inserted into the Nos3 genomic locus to target exon 2 of Nos3 by Cre-mediated excision. To generate eNOS$^{inv/inv}$ mice, we inserted an inverted exon 2 of Nos3 and two additional Lox511 sites in the loxP-eNOS construct, which allowed the Cre-induced reactivation of eNOS in a tissue of interest. The plasmids were sequenced, linearized, and electroporated in A9 ES cells (hybrid C57/129), 300 clones picked, and positive clones were screened by Southern Blot at 5' arm and by long-range polymerase chain reaction (PCR). Homozygous eNOS$^{flox/flox}$ mice or eNOS$^{inv/inv}$ mice were crossed with Cdh5-Cre/ERT2$^{pos}$ mice to obtain eNOS$^{flox/flox}$ Cdh5-Cre/ERT2$^{pos}$ and eNOS$^{flox/flox}$ Cdh5-Cre/ERT2$^{neg}$ mice or eNOS$^{inv/inv}$ Cdh5-Cre/ERT2$^{pos}$ and eNOS$^{inv/inv}$ Cdh5-Cre/ERT2$^{neg}$ mice; to induce endothelial-specific activation of the Cre-recombinase, we treated Cre positive and negative mice of each line with tamoxifen (33 mg/kg/day) for 5 consecutive days and allowed a 21 days waiting period after the last injection. Homozygous eNOS$^{flox/flox}$ mice or eNOS$^{inv/inv}$ mice were also crossed with erythroid-specific Hbb-Cre$^{pos}$ mice, to obtain erythroid-specific eNOS KO mice (eNOS$^{flox/flox}$ Hbb-Cre$^{pos}$ = RBC eNOS KO) and their respective WT littermate control (eNOS$^{flox/flox}$ Hbb-Cre$^{neg}$) or erythroid-specific eNOS KI mice (eNOS$^{inv/inv}$ Hbb-Cre$^{pos}$ = RBC eNOS KI) and their WT littermate control ( eNOS$^{inv/inv}$ Hbb-Cre$^{neg}$). In addition, eNOS$^{flox/flox}$ mice were crossed with DelCre mice to create global eNOS KO.

### Analysis of tissue-specific genetic recombination.

The Cre recombinase-dependent genetic locus recombination was determined by extracting genomic DNA from targeted and non-targeted tissues and analyzed by real-time PCR with specific primers and probes designed to recognize the floxed allele and the allele with targeted deletion (Transnetyx, Cordova, TN). For data analysis, a threshold was defined by using tissues from global eNOS KO mice obtained by the same floxed construct.

**Blood collection and blood count**

Blood and organs were collected as described previously[22]. Briefly, mice were anesthetized with isoflurane (2.0%) and killed by exsanguination. Blood was transferred immediately into tubes containing EDTA solution to reach a final concertation of 5 mM EDTA and used for blood counts in a Coulter counter. For obtaining RBCs, blood was centrifuged immediately for 10 min at 800g. Organs were harvested after 1 min of perfusion with cold PBS pH 7.4, blotted dry on filter paper, weighed, snap frozen in liquid nitrogen, and kept at −80°C until later analysis.

**Isolation of primary cells from mouse tissues by magnetic separation.**

ECs ($CD31^+$ $CD45^-$) were isolated from lung homogenates of EC eNOS KO and RBC eNOS KO mice by using magnetic anti-CD31 and anti-CD45 microbeads according to the manufacture's protocol (Milteny Biotec, Bergisch Gladbach, Germany). Lungs were explanted aseptically, incubated with an enzymatic mixture of collagenase/DNAse I for 30 min at 37°C, and homogenized by using the GentleMACS according to the manufacturer's protocol. After erythrocyte lysis with ammonium chloride solution, the cell suspension was passed through a 70 µm cell strainer and washed by gentle centrifugation. ECs were extracted by two independent steps of magnetic separation consisting of a negative selection using anti-CD45 microbeads and a positive selection using anti-CD31 microbeads. The purity and yield of cells were determined by flow cytometry analysis using specific antibodies according to standard procedures. The percentage of $CD31^+$ cells obtained by this method was a minimum of 95%. Pellets were snap frozen in RNA later.

Erythroid cells ($Ter119^+$ $CD71^+$ $CD45^-$) were isolated from the bone marrow of RBC eNOS KI mice and CondKO mice. Bone marrow was isolated from the tibia and femur (4 bones/mouse) by putting the bone in a 0.5 ml tube with a small hole in the bottom, mounted on a 1.5 ml, and centrifuged max speed for 1 min. The bone marrow was resuspended in erythrocyte lysis buffer, incubated for 10 min on ice, and filtered through a 40 µm cell strainer. The obtained cell suspension was resuspended in separation buffer (5 % bovine serum albumin in PBS with 2 mM EDTA) and incubated with anti-CD45 microbeads and an FcR receptor blocker. Magnetic separation was carried out two times to remove all $CD45^+$ cells. Afterward, $Ter119^+$ cells were separated by positive selection with anti-Ter119 microbeads incubated in the presence of an FcR receptor blocker. The purity and yield of cells were determined by flow cytometry analysis using specific antibodies against CD71, Ter119, and CD45 according to standard procedures. The percentage of $Ter119^+$ $CD71^+$ $CD45^-$ cells obtained by this method was a minimum of 92%. Pellets were snap frozen in RNA later.

**Analysis of eNOS expression in isolated cells and tissues by real-time RT-PCR**

Extraction of total RNA, retrotranscription (RT), pre-amplification, and real-time PCR from EC or mouse tissues (aorta, heart, bone marrow) was carried out by using commercial kits accordingly to the manufacturer's instructions. Briefly, cells or tissues were lysed and homogenized by using Tissue Raptor in RLT Buffer (Qiagen, Hilden, Germany) containing 2-mercaptoethanol. RNA was extracted with RNAeasy with DNAase I digestion (Qiagen) or with Ambion DNase I (Thermo Scientific, Waltham, MA, USA). RNA concentration and quality were assessed by using a NanoDrop spectrophotometer (Thermo Scientific) and RNA NanoChip Agilent 2100 Bioanalyser (Santa Clara, CA, USA) respectively. After RT with QuantiTec reverse transcription kit (Qiagen), cDNA samples from EC or erythroid cells underwent a pre-amplification step with SssoAdvacend PreAmp Supermix, followed by real-time PCR by using TaqMan Fast Advanced Master Mix (Thermo Fisher).

**Determination of eNOS expression by immunotransmission electronic microscopy**

The immunoTEM was performed as previously described[21]. Briefly, RBCs were isolated by cardiac puncture and fixed in 4% PFA plus 0.05% glutaraldehyde. RBCs were spun down, embedded in LR White, and sectioned at 70 nm sections. Rabbit eNOS Ab (Abcam cat#ab199956) was added at 1:50 overnight, with donkey anti-rabbit secondary gold beads (18 nm; Jackson Labs cat#711-2125-152) used to resolve protein localization on the RBCs.

**Determination of eNOS expression in RBC membrane preparations (ghosts) by immunoblotting and ELISA**

RBC membranes (ghosts) were prepared from 250-350 µl RBC pellet washed 3 times at low speed. RBCs were suspended at 40% Ht into a cold hypotonic buffered solution (PBS diluted 1:27) and incubated on ice (mixed with ethanol) at 0-2°C for 30 min. The ghosts are centrifuged 10 min at 20,000 g at 4° and then carefully resuspended in the starting volume of hypotonic buffered solution for 20 min. After centrifugation, the red ghosts were snap frozen in liquid nitrogen and kept at -80°C until use. Ghosts were lysed in 2 volumes of RIPA lysis buffer (1% NP40, 0.5% sodium deoxycholate, 0.1% SDS in PBS pH 7.4) with protease inhibitors (Complete, Roche) for western blotting. For ELISA ghosts were lysed with 2 volumes of commercial lysis buffer provided in the kit and ELISA was carried out according to the manufacturer's protocol (Abcam, Cambridge, UK). The total protein concentration of the supernatant was determined by the Lowry assay (Biorad).

**Determination of eNOS expression in tissues by Western blotting and ELISA**

Western blot analysis and detection were carried out as described[22]. Briefly, organs were lysed in RIPA buffer (1% NP40, 0.5% sodium deoxycholate, 0.1% SDS in PBS pH 7.4) containing a cocktail of protease and phosphatase inhibitors (Roche), homogenized at 4°C by using Tissue Ruptor (Qiagen, Hilden, Germany), sonicated for 3 min at 4°C and centrifuged at 4000 x g for 10 min at 4°C. The total protein concentration of the supernatant was determined by the Lowry assay (Biorad). Samples were loaded on 10% Bis-Tris gel (Roth) and transferred onto nitrocellulose membrane (Amersham Biosciences, Munich, Germany). The membranes were blocked for 1 hour with 5 % Milk(Bio-Rad, Düsseldorf, Germany) in T-TBS (10 mM Tris, 100 mM NaCl, 0.1% Tween) and incubated overnight at 4 °C with a mouse anti-eNOS (1:100), or monoclonal mouse anti-α-tubulin (1:5000, #T6199, Sigma-Aldrich, Schnelldorf, Germany) in T-TBS. After washing for 1 hour in T-TBS, the membranes were incubated with HRP-conjugated goat anti-mouse or anti-rabbit secondary antibodies (1:5000; BD Biosciences, St Hose, CA) and bands were detected using West Pico Chemiluminescence Detection Reagent and ChemiDoc (Biorad).

**Determination of eNOS expression in RBC lysates by immunoprecipitation/immunoblotting.**

Lysis and extraction of eNOS from RBCs were carried out by immunoprecipitation by using magnetic beads cross-linked to an antibody anti-eNOS (custom made from #624086 anti-eNOS/NOS type III antibody, stock: 1 mg/ml in PBS pH 7.4, BD Bioscience, Erembodegem, Belgium BD Bioscience), as described[8]. Briefly, RBCs were lysed with 2 volumes of RIPA buffer and the total protein concentration of the supernatant was determined by the Lowry assay. The lysate was diluted to a concentration of 100 µg/1ml and incubated overnight with Epoxy-Dynabeads crosslinked with an antibody anti-eNOS; after magnetic separation, proteins were eluted with LDS sample buffer and loaded on a 6-12 % Tris-Acetate pre-cast gels (Invitrogen) and transferred onto nitrocellulose (Amersham Biosciences, Munich, Germany). The membranes were blocked for 1 hour with 5 % Milk (Bio-Rad, Düsseldorf, Germany) in T-TBS (10 mM Tris, 100 mM NaCl, 0.1% Tween) and incubated overnight at 4 °C with a mouse anti-eNOS (1:100) in T-TBS. After washing for 1 hour in T-TBS, the membranes were incubated with HRP-conjugated goat anti-mouse or anti-rabbit secondary antibodies (1:5000; BD Biosciences, St Hose, CA) and bands were detected using West Fento Chemiluminescence Detection Reagent and ChemiDoc (Biorad).

**Measurement of blood pressure and systemic hemodynamics**

Invasive assessment of hemodynamic parameters was carried out by using a 1.4 F Millar pressure-conductance catheter (SPR-839, Millar Instrument, Houston, TX, USA) placed into the left ventricle through the right carotid artery according to the closed chest method as described[22]. The pressure was recorded by a Millar Box and analyzed with LabChart 7 (AD Instruments, Oxford, UK) to assess LV developed pressure, rate of pressure development

($dP/dt_{max}$), and rate of pressure decrease ($dP/dt_{min}$)[22]. For assessment of hemodynamic responses in awake mice, we used radiotelemetry. Mice were surgically implanted under temporary anesthesia with ketamine/xylazine with a microminiaturized electronic monitor (PA-C10; Data Sciences International (DSI); St. Paul, MN, USA) attached to an indwelling aortic catheter. Digitized hemodynamic data were continuously sensed, processed, and transmitted via radio frequency signals to a nearby receiver (acquisition period: 3 min every hour). Mice were allowed at least a 5-day post-surgery stabilization period on a standard diet and drinking water before starting the acquisition of hemodynamic data. A scheme of the experimental protocol is depicted in **Fig. 3 C, D**. After 3 days of baseline measurements, we measured hemodynamic responses to NOS inhibition by the administration of N(gamma)-nitro-L-arginine methyl ester (L-NAME, 1 mg/ml in drinking water for 3 days; we determined that mice drink 5 ml/day independently on the presence of L-NAME, thus resulting in a dose of 166 mg/Kg/day); these were followed by hemodynamic responses to increased L-Arginine bioavailability achieved by administration of the arginase inhibitor N-Hydroxy-nor-L-arginine (NorNOHA, i.p. 10 mg/Kg for 3 days).

### Measurement of endothelial function/vascular reactivity *ex vivo*

Thoracic aortas were excised and their functional reactivity analyzed in an organ bath as previously described[23]. Briefly, after an equilibration phase of 60 min, aortic rings were constricted twice by the administration of 80 mM KCl. The second vasoconstriction was taken as the maximal receptor-independent vasoconstriction. Endothelial function was assessed by the administration of cumulative doses of acetylcholine (ACh; 0.1 nM-10 µM). After washing, vascular contractility was determined by applying cumulative doses of the $\alpha_1$-adrenergic receptor agonist phenylephrine (PE; 0.1 nM-10 µM). Subsequently, NO-dependent vasodilation was induced by applying a cumulative dose of sodium nitroprusside (SNP; 0.01 pM-10 µM). The half-maximal effective concentration ($EC_{50}$) of PE and ACh were determined in preliminary experiments.

### Measurement of vascular function *in vivo*

Vascular function was measured as flow-mediated dilation (FMD) with a Vevo 2100 with a 30-70 MHz linear array microscan transducer (VisualSonics) as described[22]. The method consists of the analysis of changes in vessel diameter of the iliac artery in response to shear stress after vascular occlusion by using a cuff. During the experiment, mice were kept under 1.5-2% isoflurane anesthesia, a heart rate of 400-500 bpm, a breathing rate of about 100 breaths/min, and a 37°C body temperature.

### Determination of NO metabolites in blood and tissues

Nitrosated (S-nitroso and N-nitroso) products (RXNO), and nitrosylheme (NO-heme) were quantified by gas phase chemiluminescence as described[24]. For nitrite and nitrate analysis, samples were deproteinized with ice-cold methanol (1:1 v/v), cleared by centrifugation, and subjected to analysis by high-performance liquid chromatography using a dedicated nitrite/nitrate analyzer (ENO20, Eicom, Japan)[25].

### Vessel staining

Sections were deparaffinized and rehydrated followed by heat-mediated antigen retrieval using citric buffer, pH 6.0 (Zytomed Systems, Cat. No. ZUC028-500). Endogenous streptavidin and biotin were blocked by a Streptavidin/Biotin Blocking Kit (Vector Labs, Cat. No SP-2002). ECs were stained with biotinylated GSL I isolectin B4 (Vectorlabs, Cat. No. B-1205, 1:50 dilution) at 4° C overnight. Streptavidin-Cy5 (ZyMax Grade, Invitrogen, Cat. No. 438316, dilution: 1:50) was used for detection. Smooth muscle cells were stained with anti-alpha-smooth muscle actin (SMA) -Cy3 at room temperature (Sigma Aldrich, Cat. No.C6198, dilution: 1:200). Sections were mounted with Roti-Mount FluorCare DAPI (Roth, Karlsruhe, Germany).

**Statistical analysis**

Sample size was calculated a priori by using G-Power V.3.1 (Heinrich Heine University of Düsseldorf). Statistical analysis was carried out with GraphPad Prism 9 for macOS (Version 9.0.2(134)). Unless stated otherwise, the results are reported as means ± standard deviation (SD). Normal distribution was tested by the D'Agostino-Pearson test. Comparisons among multiple groups were performed using 1-way and 2-way analysis of variance (ANOVA) or 2-way repeated measures (RM)-ANOVA, as appropriate, followed by Tukey's or Sidak's post-hoc analysis, as indicated. Where indicated, unpaired Student's t-test with Welch correction was used to determine if two groups of data were significantly different. The Mann-Whitney test was carried out when data were not normally distributed. $p < 0.05$ was considered statistically significant.

## Supplemental Tables

**Table I. Blood count parameters evaluated in EC eNOS KO and RBC eNOS KO mice.** The table summarizes the blood count parameters measured in EC eNOS KO, RBC eNOS KO mice, and respective WT controls. Abbreviations: RBC, red blood cells; HCT, hematocrit; HGB, hemoglobin; RDW, RBC distribution width; MCHC, mean corpuscular hemoglobin concentration; MCH, mean corpuscular hemoglobin; MCV, mean corpuscular volume; WBC, white blood cells; Lymph, lymphocytes; Mo, monocytes; and Gra, granulocytes; PLT, platelet count; MPV, mean platelet volume. All data are expressed as mean ± SD. T-test.

| | **WT** | **EC eNOS KO** | **p** | **WT** | **RBC eNOS KO** | **p** |
|---|---|---|---|---|---|---|
| | **$eNOS^{flox/flox}$ Cdh5-Cre/$ERT2^{neg}$ +TAM** | **$eNOS^{flox/flox}$ Cdh5-Cre/$ERT2^{pos}$ +TAM** | | **$eNOS^{flox/flox}$** | **$eNOS^{flox/flox}$ $HbbCre^{pos}$** | |
| ***n*** | 9 | 15 | | 6 | 12 | |
| | | ***Red blood cell count*** | | | | |
| ***RBC ($10^6$/µl)*** | 9.93±1.94 | 11.29±1.60 | 0.0745 | 9.26±1.65 | 9.64±1.58 | 0.6477 |
| ***HCT (%)*** | 50.78±10.11 | 56.93±8.87 | 0.1324 | 47.95±7.79 | 47.51±5.20 | 0.8902 |
| ***HGB (g/dl)*** | 14.90±3.50 | 16.75±2.75 | 0.1639 | 15.03±2.85 | 14.99±3.06 | 0.9782 |
| | | ***Red blood cell indexes*** | | | | |
| ***RDW (%)*** | 15.10±0.88 | 14.87±0.37 | 0.3720 | 16.06±1.46 | 15.14±0.61 | 0.2625 |
| ***MCHC (g/dl)*** | 29.19±2.18 | 29.41±1.34 | 0.7568 | 31.25±1.21 | 30.05±1.83 | 0.0613 |
| ***MCH (pg)*** | 14.91±1.24 | 14.87±0.67 | 0.9234 | 16.22±0.44 | 15.49±0.93 | 0.0907 |
| ***MCV (µm³)*** | 51.22±1.86 | 50.67±0.72 | 0.3068 | 52.00±0.89 | 51.50±1.57 | 0.4829 |
| | | ***White blood cell count*** | | | | |
| ***WBC ($10^3$/µl)*** | 9.73±2.20 | 6.70±0.85 | 0.1727 | 7.93±2.35 | 6.91±2.77 | 0.4498 |
| ***Lymph ($10^3$/µl)*** | 5.14±1.76 | 4.04±1.49 | 0.1231 | 5.13±2.05 | 4.34±1.71 | 0.3974 |
| ***Lymph (%)*** | 70.63±7.74 | 70.80±11.64 | 0.9700 | 65.15±12.82 | 65.36±13.90 | 0.9759 |
| ***Mo ($10^3$/µl)*** | 0.32±0.16 | 0.29±0.07 | 0.6504 | 0.40±0.25 | 0.31±0.24 | 0.4671 |
| ***Mo (%)*** | 5.00±1.78 | 5.97±1.74 | 0.2045 | 5.73±3.27 | 4.95±1.85 | 0.5212 |
| ***Gra ($10^3$/µl)*** | 2.27±0.81 | 1.99±0.61 | 0.4893 | 2.40±0.98 | 2.23±1.50 | 0.8097 |
| ***Gra (%)*** | 24.33±8.41 | 23.24±11.11 | 0.8018 | 28.95±9.80 | 29.69±103.18 | 0.9084 |
| | | ***Platelet count*** | | | | |
| ***PLT ($10^3$/µl)*** | 1134.90±479.51 | 1144.07±334.80 | 0.9575 | 1770.67±13.18 | 1570.00±348.28 | 0.1957 |
| ***MPV (µm³)*** | 5.11±0.25 | 5.34±0.33 | 0.1257 | 5.78±0.34 | 5.78±0.61 | 0.9956 |

# Supplementary Figures

## Online Figure I.

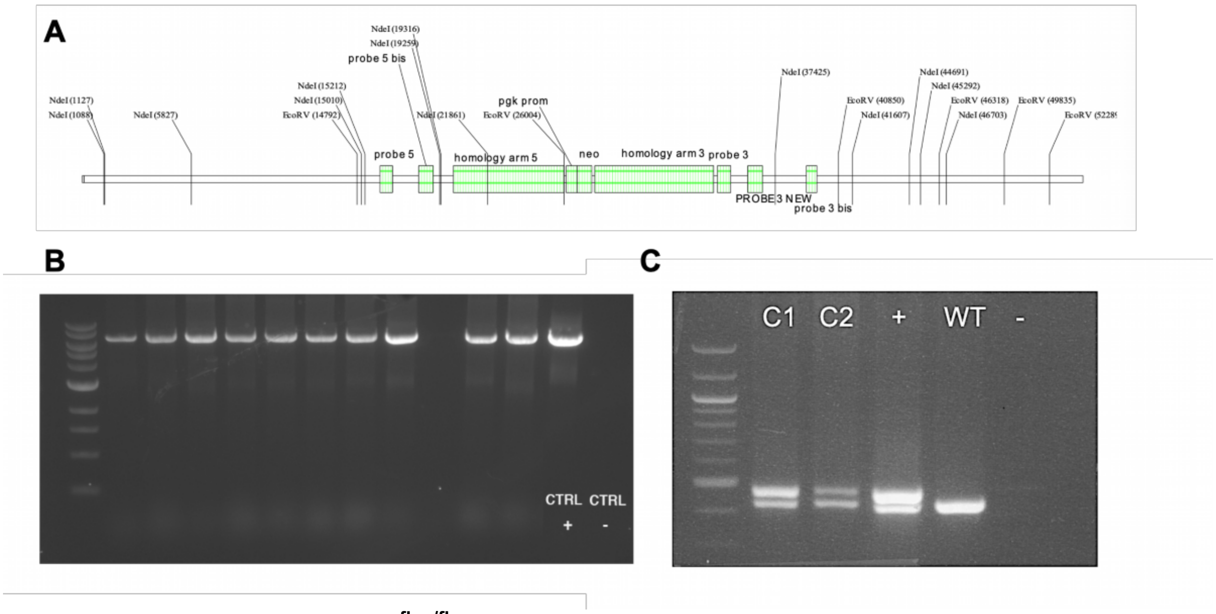


**Figure I. Generation of eNOS$^{flox/flox}$ mice**. **(A)** Schematic representation of the gene targeting construct showing the homology arms, the neomycin cassette for selection, and the positions of the probes for southern blotting analysis. **(B)** Confirmation of stable transfection of the targeting construct in ES cells as determined by long-range PCR analysis (Foreword primer (FW): 5'-CCTTCTTGACGAGTTCTTCTGAGCGGG-3'; Reverse primer: 5'-AGCTATTCTAGCATCCAGAGAGTGTGC-3'). **(C)** Genotyping of heterozygous eNOS$^{flox/flox}$ mice confirming the presence of the LoxP sequence (ATAACTTCGTATAATGTATGCTATACGAAGTTAT) detected by PCR (FW primer 5'-ATCACCTACGACACCCTCAG-3' and RW primer 5'-CCAAG GAAGTTACAGAGCCG-3').

## Figure II

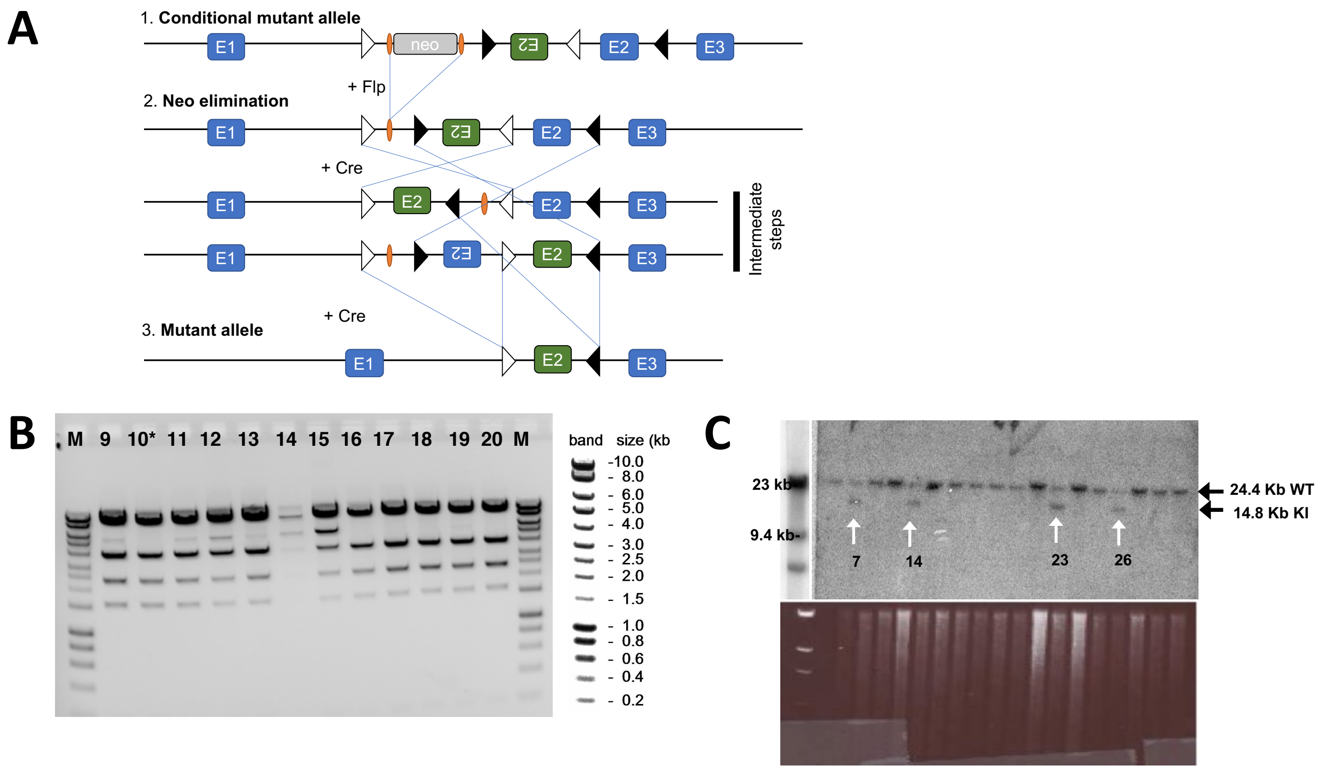


**D**

```
GGTGGTGGTGGGAAACGAGATAGTGGCTCACAGCACACATCAACTCTCTTGTACTCCCTTGCTGGGTGCGTCATTTGACCACGCCTTGAAGCCTTTAAGTGGGGGCACAGAAGGT
                           EcoRV                             loxP
ACTGCCCACCTCCTAAGGCTGTTGTGAgatatcaattaaccctcactaaagggcggccgcataacttcgtataatgtatgctatacgaagttatGAGCTGACAGAAAGTGTCAGG
AAGAGGAGGGTGGGCAACGACCCTGTAGGGTTACTGATGCCGTACAGTGCTGCCCTTGAACAGTGATGGGGAGGCCTAAAGGAGACAAACTTCTCACCCATGACTCTATGCATTC
             exon2
CTGGCCCACAACAGCCCCCCGCTAACCCGGCCCCCAGACGGACCCAGGTTTCCTCGAGTAAAGAATTGGGAAGTGGGCAGCATCACCTACGACACCCTCAGTGCCCAGGCTCAGC
AGGTAAGGACAGGTCCTGCCCGTGTTTCTAGGCCTGGTCCCAGATCAGGACCCAAACCAAGAAACTCCACTTTGTTCACATTTGGCCCTTGTCTGCAGAGAGTAGTGAAAGCCAG
                                     lox511                          MunI
GAAGGACCAGAGGGATCAAGGGGCATTGCAAGAACTTataacttcgtatagtatacattatacgaagttatcaattgCAGCAAGCCTTTGGCCCTGAGCCCCAAGCCCTAAGCAA
CCAATCTCATCCCATTTCCATTATCAGCCCTGCTCTGAGCCCTTGCTTTCCCGAGCCCGCCTCCCTCTGCCCTTGCTAACTAACCACAGCTCACTCATCCTGTACTATGACCTGA
                                exon3
TCCTGGCCTTTGTGTCCCCTGTTCTGGTTCAGGATGGGCCCTGTACCTCAAGACGCTGCTTGGGATCCCTGGTGTTTCCAAGGAAGTTACAGAGCCGGCCCACCCAGGGCCCTTC
ACCCACTGAGCAGCTATTGGGTCAAGCCCGGGACTTCATCAATCAGTACTATAACTCCATCAAAAGGTGGGTCCCCT
```

**Figure II. Generation of eNOS$^{inv//inv}$ mice and sequence of Cre-induced inversion of exon 2.** (**A**) Schematic representation of the gene targeting construct showing the neomycin resistance cassette for clone selections and the inverted exon 2. The gene targeting construct for generating eNOS$^{inv//inv}$ was obtained by Cre-induced removal of exon 2 and insertion of the FRT-neo-FRT-LoxP resistant cassette and a synthetic DNA fragment containing an orphan loxP site, the lox 511 sites, and the inverted exon 2. (**B**) Analysis of clones obtained after retransformation by restriction digestion with *Xba*I. Expected banding pattern: 9006 bp, 3796 bp, 2287 bp, 1550 bp and 194 bp (target construct), 9006 bp, 5524 bp, 194 bp (unmodified plasmid). M: DNA size marker. Amplification products were analyzed on a 1% (w/v) agarose gel. The clone selected for retransformation is marked with an asterisk (*). Plasmid DNA isolated from religation-retransformation clone #10 was finally subjected to sequence analysis with primers GBPR14, GBPR16, GBPR28, GBPR75, GBPR91, and GBPR135. Sequencing confirmed base-pair-precise modification of the *Nos3* genomic locus and integrity of all functional elements. (**C**) Confirmation of stable transfection of the targeting constructs in ES cells as determined by southern blotting (**D**) Sequence of the mutant allele after Cre-induced inversion of exon 2.

Figure III

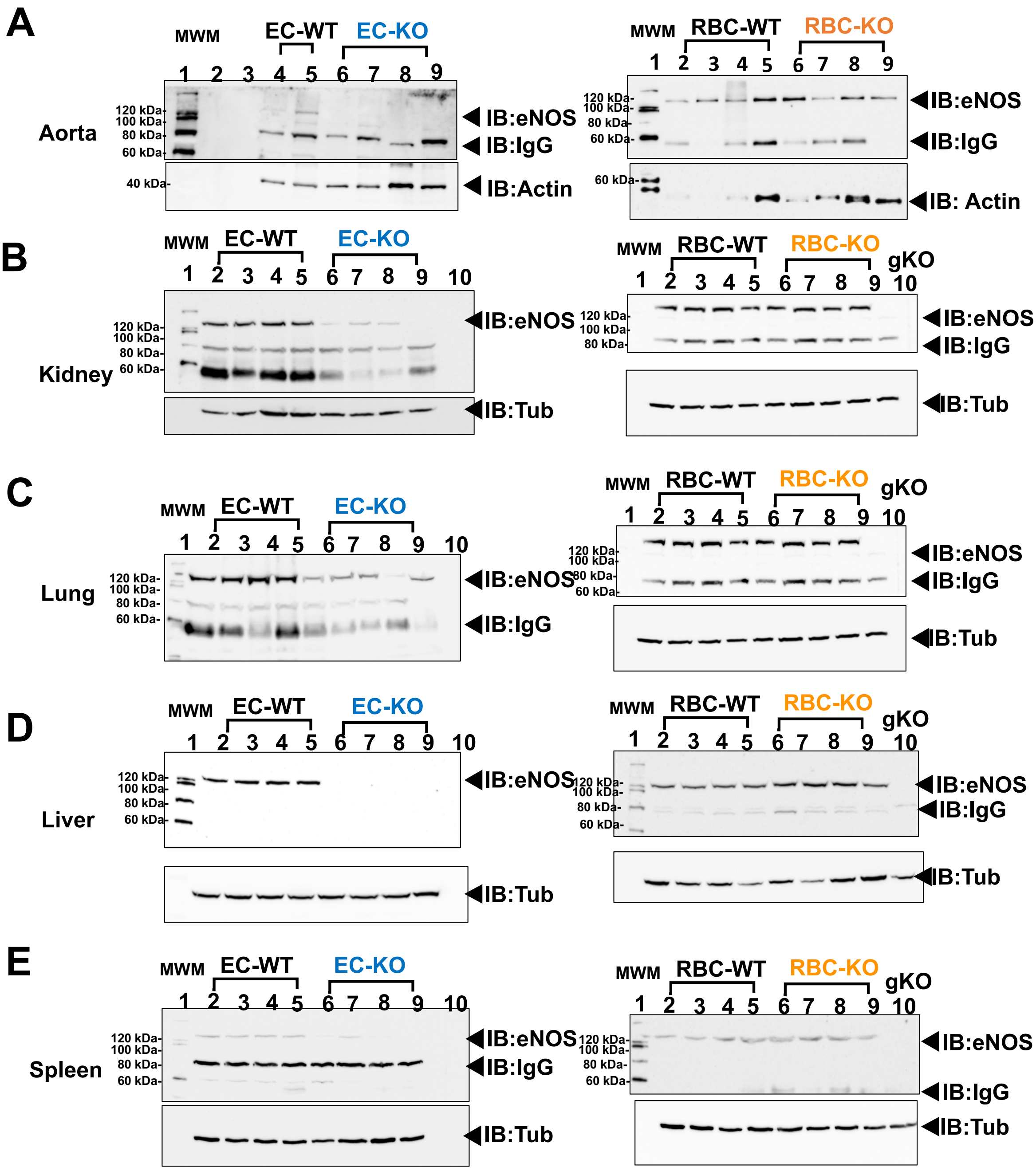


**Figure III. Expression of eNOS is significantly decreased in multiple tissues of EC eNOS KO and RBC eNOS KO, as compared to their WT littermate mice.** Organs from the same 8 mice were lysed in RIPA buffer + protease inhibitors and analyzed by immunoblotting. **Left panel:** 4 WT (eNOS$^{flox/flox}$ Cdh5-Cre/ERT2$^{neg}$ + TAM) and 4 EC eNOS KO (eNOS$^{flox/flox}$ Cdh5-Cre/ERT2$^{pos}$ + TAM)**. Right panel:** 4 WT (eNOS$^{flox/flox}$ HbbCre$^{pos}$ ) and RBC eNOS KO (eNOS$^{flox/flox}$ HbbCre$^{neg}$ (**A**) 10 µg aorta lysate; note: lanes 2 and 3 are empty as aorta lysate of these two mice were not available. (**B**) 100 µg kidney lysate. (**C**) 100 µg lung lysate. (**D**) 100 µg of liver lysate. (**E**) 100 µg of spleen lysate. IB: eNOS, immunoblot of eNOS (135 kDa); IB: IgG (IgG heavy chain of 80 and 60 KDa); IB: Actin (45 kDa), loading control; IB: Tub, Tubulin (55 kDa), loading control.

Figure IV

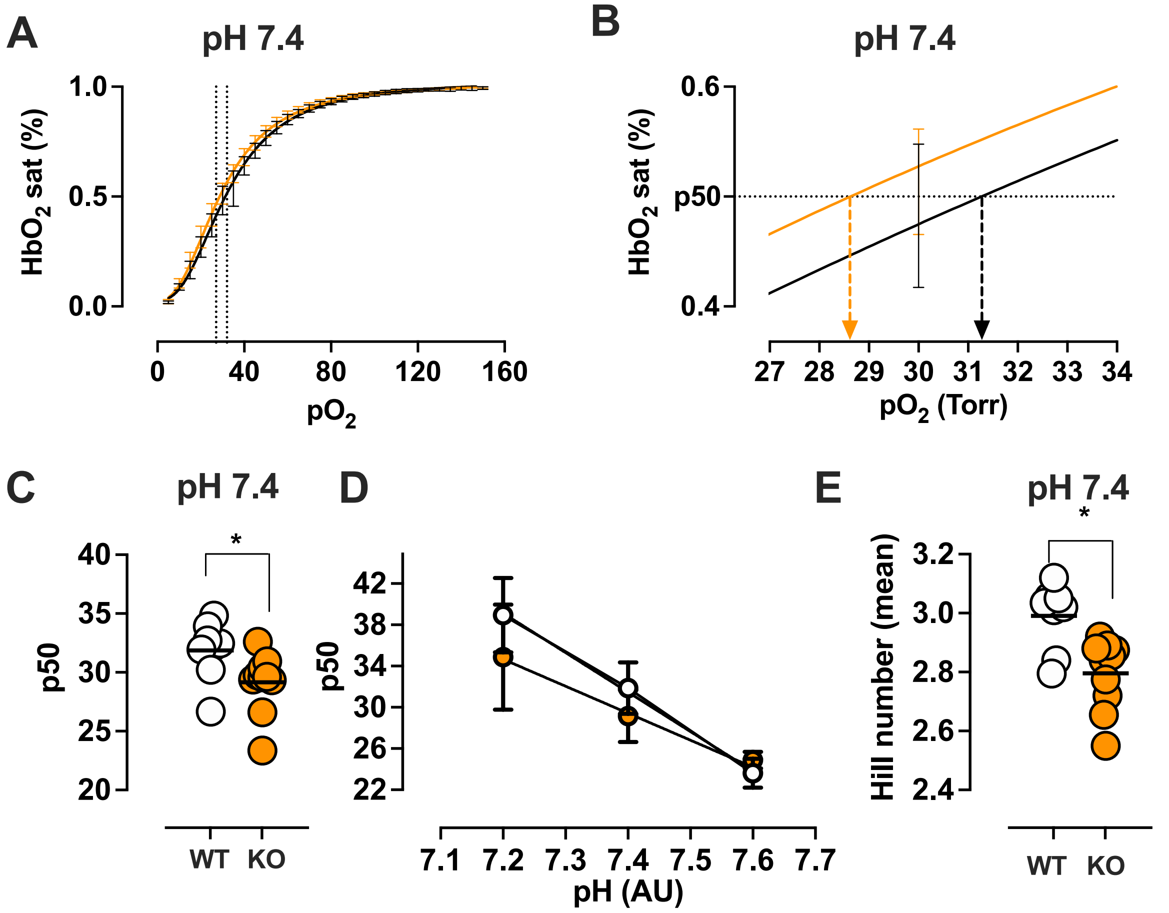


**Figure IV. Oxygen affinity and bonding cooperativity in RBCs from RBC eNOS KO mice and WT mice.** Oxygen affinity was significantly higher and oxygen-binding cooperativity was significantly lower in RBCs from RBC eNOS KO (orange) mice as compared to WT mice; this difference progressed as pH was lowered within the physiological range (from 7.2 to 7.6). Significant differences were found at pH = 7.4. (**A**) Percentage hemoglobin saturation at pH 7.4 at increasing oxygen partial pressure. (**B**) Curves between 27 and 34 Torr. (**C**) p50 at pH 7.4; t-test *p <0.05. (**D**) Bohr plot (**E**) Hill number at pH 7.4; t-test *p <0.05.

**Figure V**

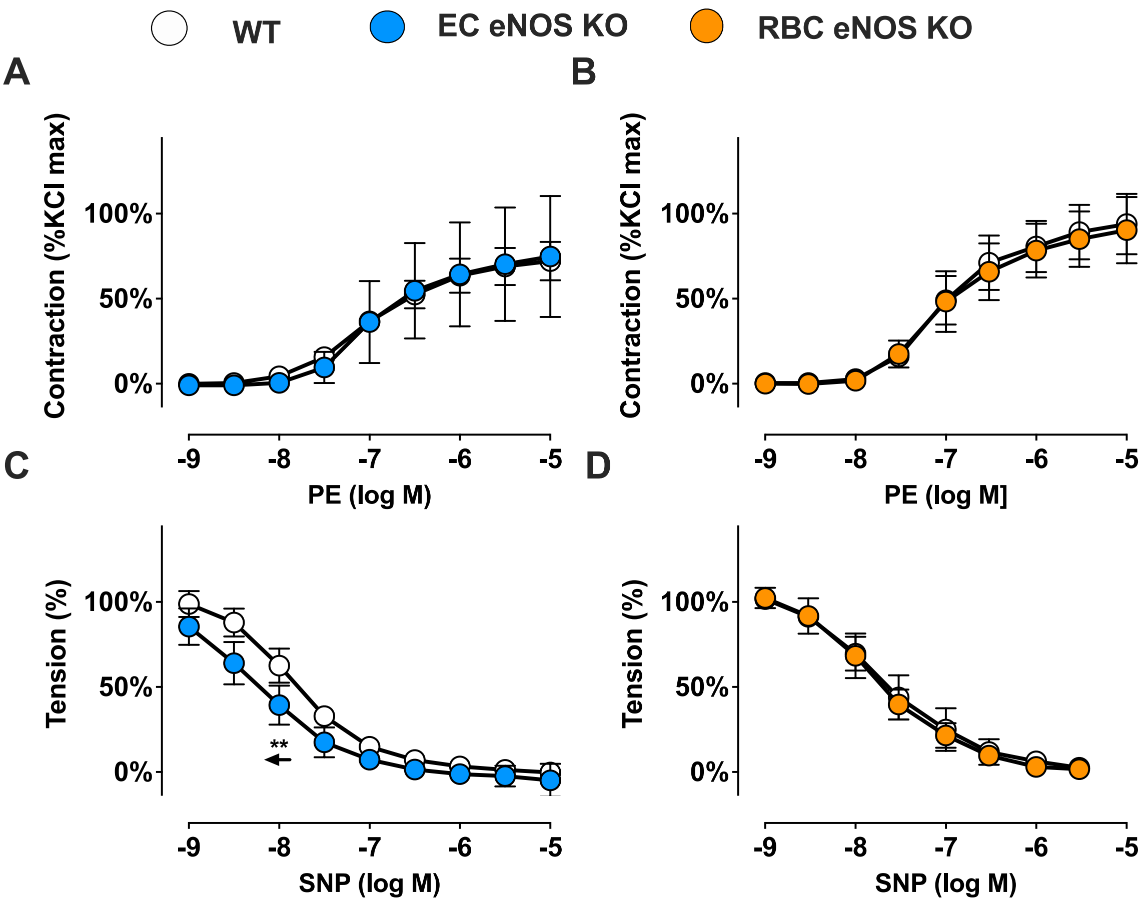


**Figure V. Vascular endothelial function of aortic rings from EC eNOS KO and RBC eNOS KO mice (SNP and PE responses). (A, B)** The contractile response of aortic rings to increasing concentrations of phenylephrine (PE) is not different in EC eNOS KO **(A)** or RBC eNOS KO **(B)**, as compared to their WT littermate controls. (**C, D**) The vasodilatory response of aortic rings to increasing concentrations of the NO donor sodium nitroprusside (SNP) is increased in EC eNOS KO and fully preserved in RBC eNOS KO mice, as compared to their littermate WT controls. 2-way ANOVA $p<0.0001$; Sidak's $^{**}p<0.01$ $10^{-9}$ > SNP > $10^{-7.5}$ M

Figure VI

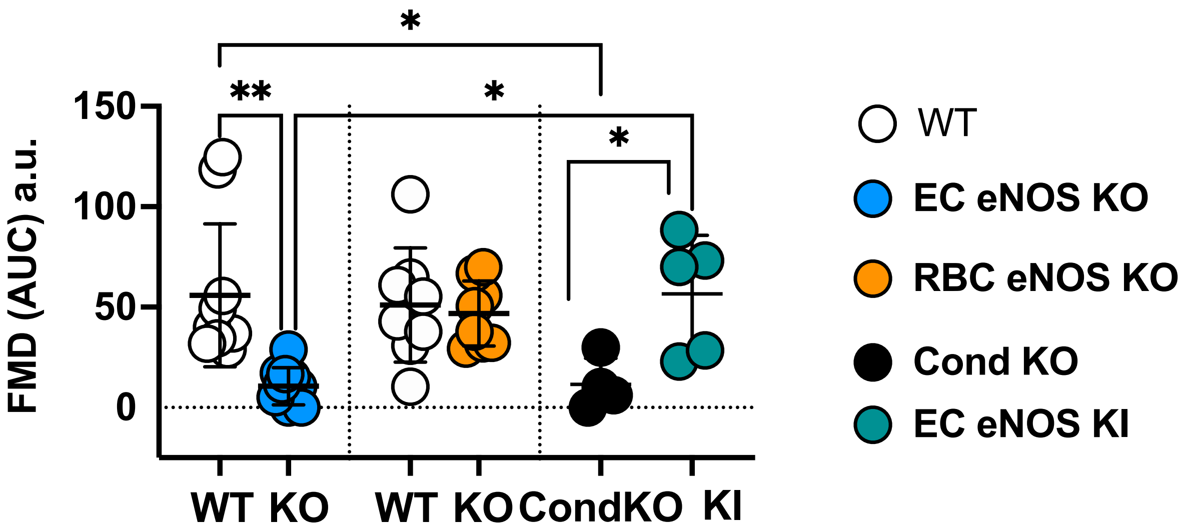


**Figure VI. The flow-mediated dilatory response is impaired in EC eNOS KO and CondKO and fully preserved in RBC eNOS KO, EC eNOS KI as compared to WT controls.** The total response to flow-mediated dilation was quantified as the area under the curve. Please refer to Figure 2 and Figure 5 in the main manuscript.1-way ANOVA $p<0.001$; Tukey *$p<0.05$; **$p<0.01$.

**Figure VII**

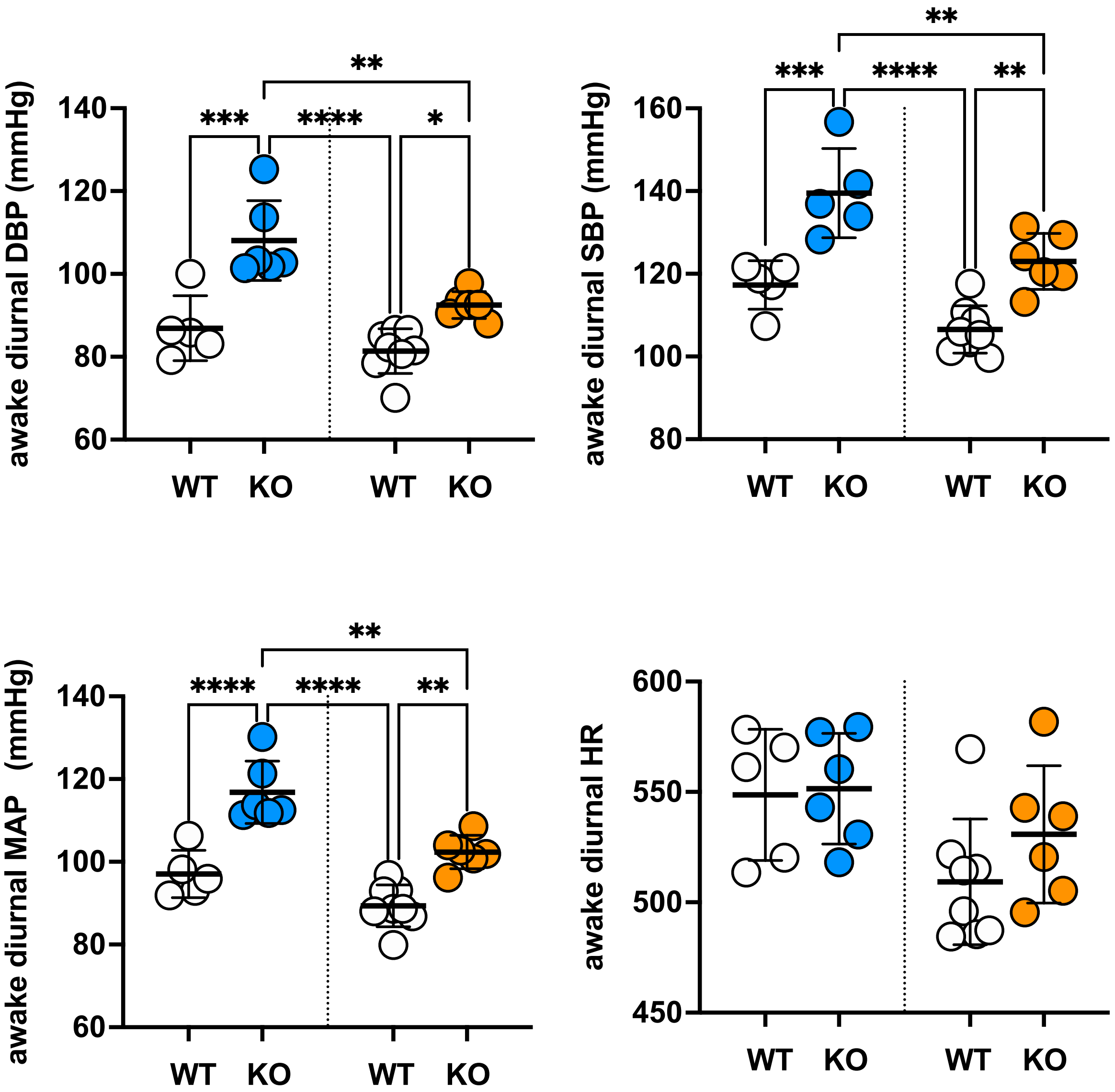


**Figure VII. Diurnal hemodynamic parameters as determined by radiotelemetry in EC eNOS KO, RBC eNOS KO, and respective WT littermate mice**. The comparison of hemodynamic parameters determined in awake mice by radiotelemetry with the same parameters determined under anesthesia by Millar catheterization (please refer to Table 1 and Table 2 in the main manuscript) shows that anesthesia induces a reproducible decrease in SBP; DBP and MAP in all parameters and a corresponding increase in HR in the WT groups. Overall, the differences among the WT/KO groups in awake mice by telemetry are statistically significant and qualitative the same as the measurements obtained in narcosis.1-way-ANOVA $p<0.001$ Tukey’s test $^{**}p<0.01$; $^{***}p<0.001$; $^{****}p<0.0001$.

**Figure VIII**

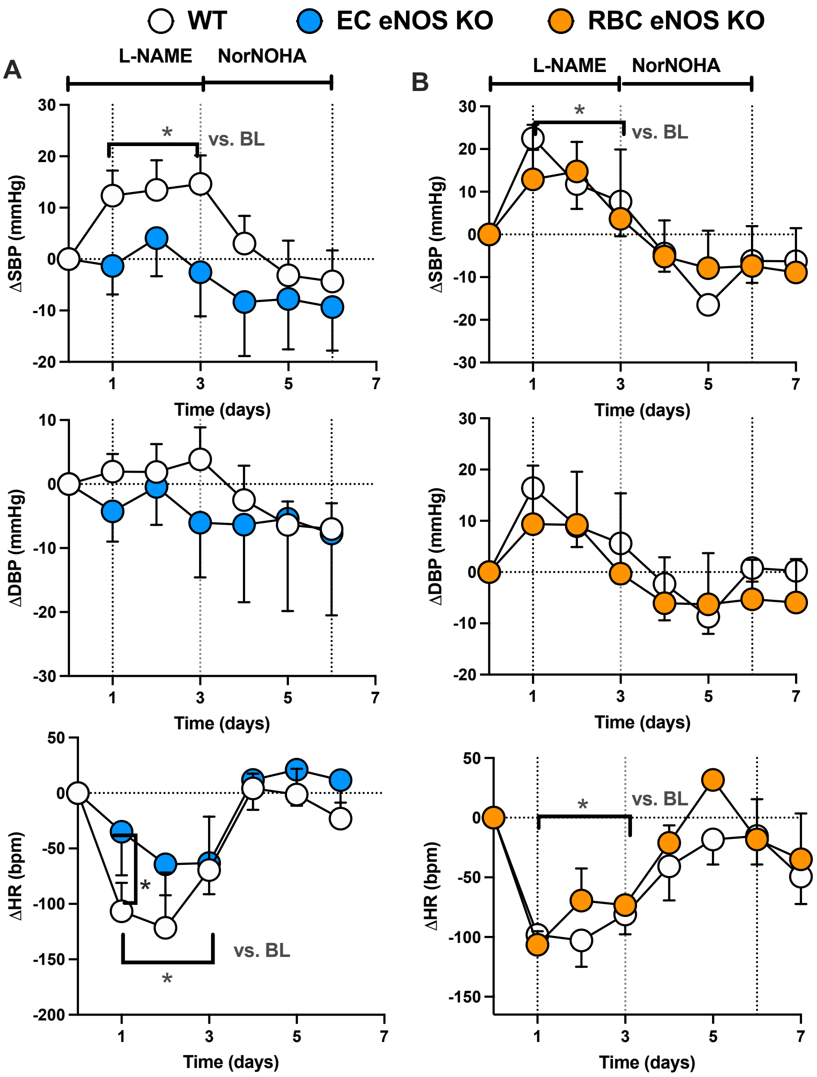


**Figure VIII. Effects of pharmacological modulation of eNOS in awake EC eNOS KO and RBC eNOS KO on blood pressure changes.** (**A**) Radiotelemetric measurements of changes in systolic BP (SBP), diastolic BP (DBP), and HR in awake EC eNOS KO mice (n = 7) show that NOS inhibition by L-NAME or increase in arginine availability by the administration of the arginase inhibitor NorNOHA did not affect SBP in awake EC eNOS KO mice (blue) as compared to WT mice (n = 5). 2-way RM ANOVA p<0.001; Holm-Sidak's *p<0.05 vs. baseline. (**B**) Radiotelemetric measurements of changes in SBP, DBP, and HR in a sub-cohort of awake RBC eNOS KO mice (n = 5) show that treatment with L-NAME further increases SBP in RBC eNOS KO and WT littermate mice (n = 3) to the same extent as in the WT controls (although the baseline levels between RBC eNOS KO and WT controls were significantly different, see **Figure 3C, D**); increase of arginine bioavailability by the administration of NorNOHA rapidly restored BP to the baseline levels. 2-way RM ANOVA p<0.001; Holm-Sidak's *p<0.05 vs. baseline

## Figure IX

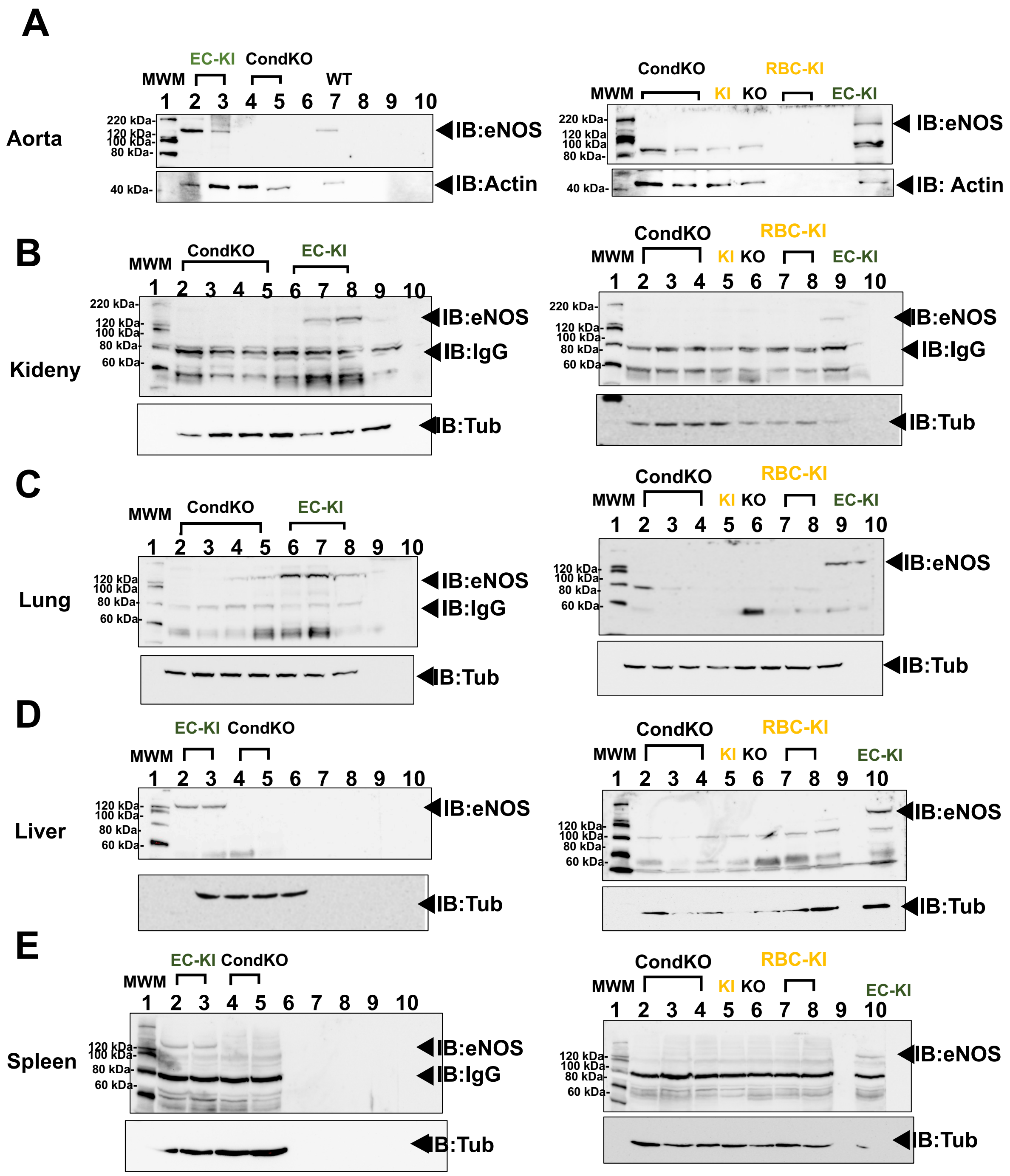


**Figure IX. Reactivation of eNOS expression in multiple tissues of EC eNOS KI mice and lack of eNOS expression in tissues from RBC eNOS KI and CondKO mice.** Organs from the same mice were lysed in RIPA buffer + protease inhibitors and analyzed by immunoblotting. **Left panel:** 4 CondKO (eNOS$^{inv/inv}$ Cdh5-Cre/ERT2$^{neg}$ + TAM) and 3 EC eNOS KI (eNOS$^{inv/inv}$ Cdh5-Cre/ERT2$^{pos}$ + TAM)**. Right panel:** 4 CondKO (eNOS$^{inv/inv}$ HbbCre$^{neg}$ ) and RBC eNOS KI (eNOS$^{inv/inv}$ HbbCre$^{pos}$ ). Please note for some mice we did not have all samples available and lanes were left empty. (**A**) 10 µg aorta lysate; note: lane 7 is a WT (eNOS$^{flox/flox}$). (**B**) 100 µg kidney lysate; (**C**) 100 µg lung lysate; (**D**) 100 µg of liver lysate; (**E**) 100 µg of spleen lysate. **IB: eNOS**, immunoblot of eNOS (135 kDa); **IB: IgG** (IgG heavy chains are visible at 80 or 50 kDa); **IB: Actin**, Actin (45 kDa), loading control; **IB: Tub**, Tubulin (55 kDa), loading control.

**Figure X**

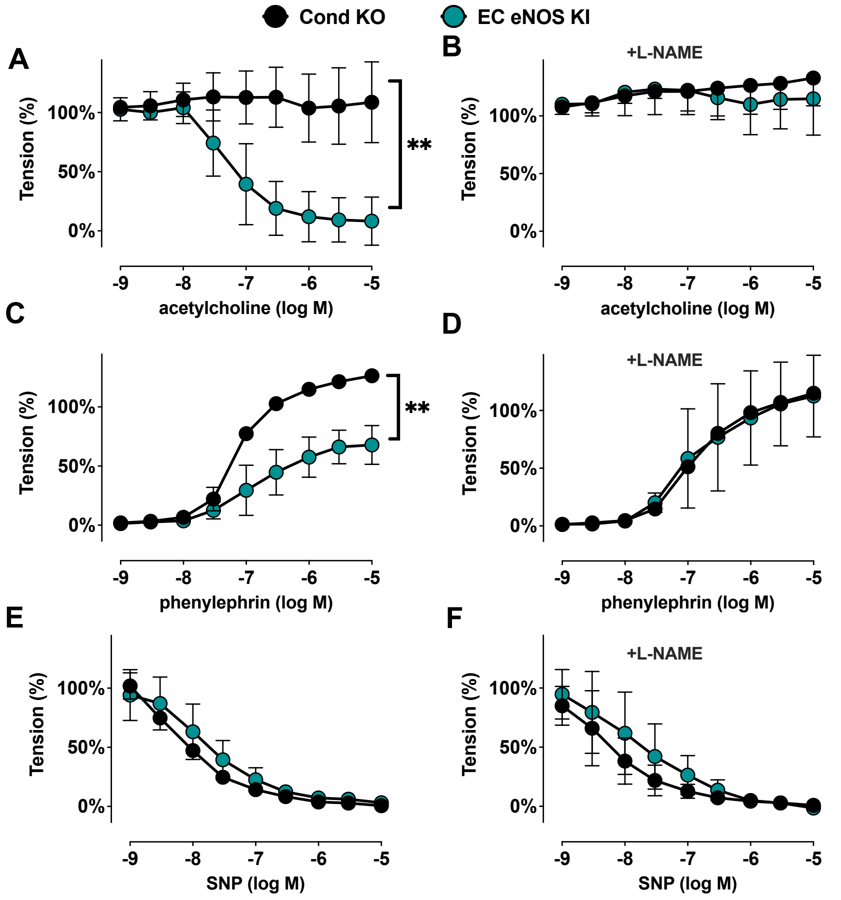


**Figure X. Vascular endothelial function of aortic rings from EC eNOS KI and CondKO littermate mice (SNP and PE responses).** (**A**) The reactivation of eNOS in the vascular endothelium fully restores the ACh response in EC eNOS KI (green) as compared to CondKO littermates controls (black). 2-way-ANOVA $p<0.0001$; Sidak's **$p<0.01$ ACh > $10^{-7}$ M. (**B**) The administration of the NOS inhibitor L-NAME fully blocked the ACh-induced vasorelaxation in EC eNOS KI mice (green). (**C**) The contractile response of aortic rings to increasing concentrations of phenylephrine (PE) is decreased in EC eNOS KI mice as compared to CondKO mice. 2-way-ANOVA $p<0.0001$; Sidak's **$p<0.01$ PE > $10^{-7.5}$ M. (**D**) The administration of the NOS inhibitor L-NAME increase PE-induced contractile response in EC eNOS KI to the level of the CondKO mice. (**E**) The vasodilatatory response of aortic rings to increasing concentrations of the NO donor sodium nitroprusside (SNP) is not significantly different from the response in littermate CondKO mice. (**F**) No difference in SNP-dependent relaxation between CondKO and EC eNOS KI after L-NAME administration.

Figure XI

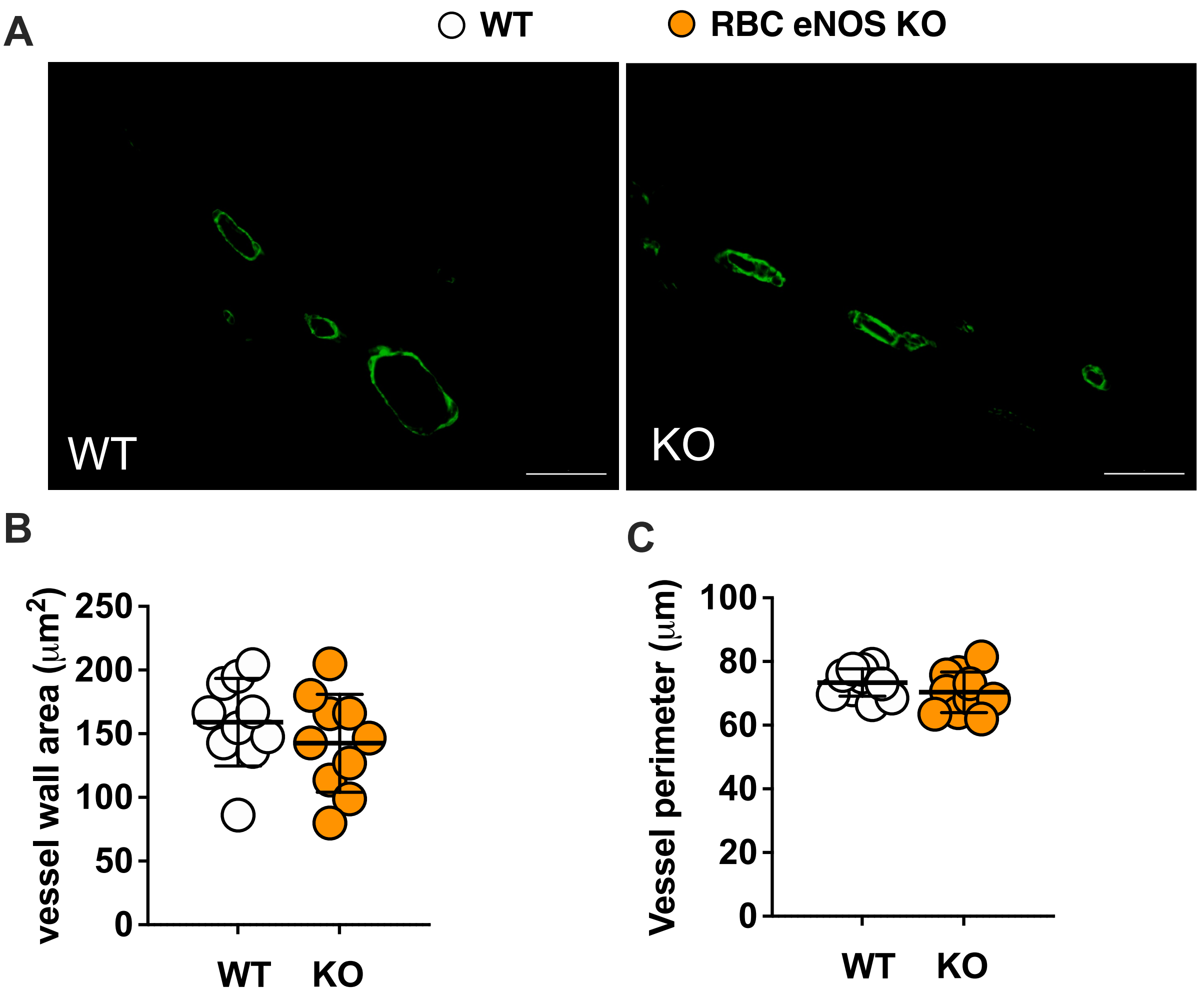


**Figure XI. Morphometry of cardiac blood vessels ranging from 50-100 µm in perimeter in RBC eNOS KO and WT mice.** (**A**) Representative picture of the histological specimen showing cardiac blood vessel of a WT and a RBC eNOS KO mouse. We determined the vessel perimeter and vessel area of 9-10 vessels per animal (n = 3 animal/groups). (**B**) Vessel perimeter in µm² ( n = 3 animal / groups). (**C**) Vessel wall area in µm². Data are shown as mean ± SD. Vessel perimeter or area was not different between RBC eNOS KO and WT mice.

**Figure XII**

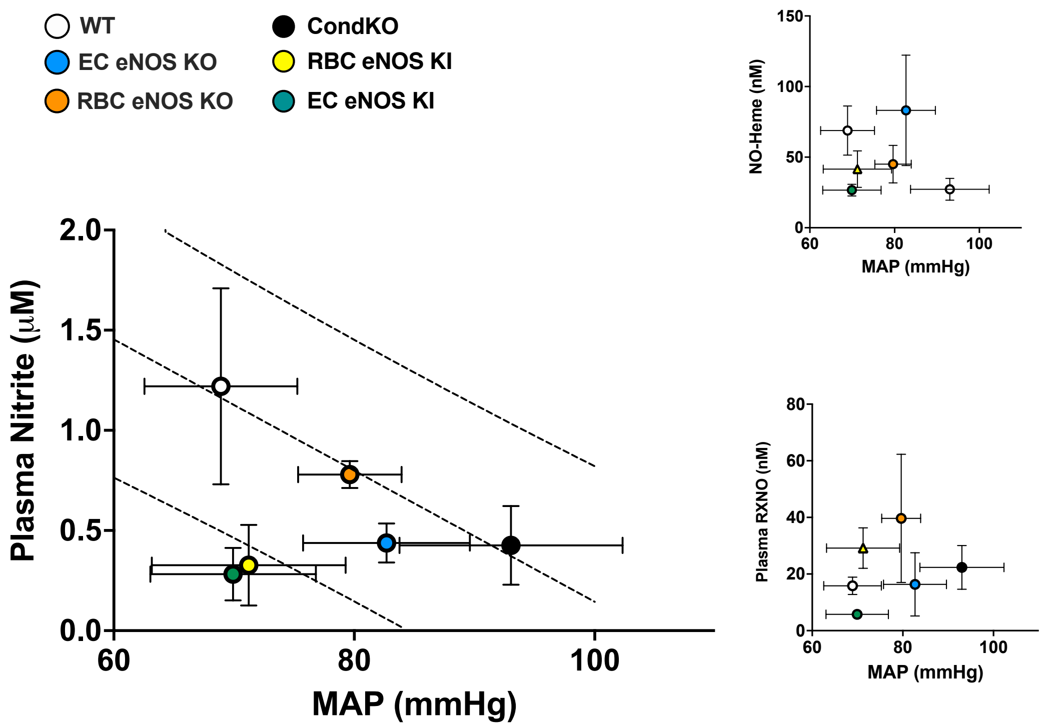


**Figure XII. Correlations of plasma nitrite and MAP.** The diagram shows the correlation between plasma nitrite levels and the MAP of all strains analyzed. Values of WT control groups and CondKO control groups were grouped for simplicity. The line shows the simple linear regression among WT, RBC eNOS KO, EC eNOS KO, and CondKO and the 95% coefficient of interval. Among these 4 groups the correlation coefficient according to Pearson was p = 0.0898 R = -0.912 $R^2$ = 0.8285. The two insets show correlation analysis between NO-heme and MAP or plasma RXNO and MAP, which was not significant.
